# Differentiation, evolution and utilization of natural alleles for cold adaptability at the reproductive stage in rice

**DOI:** 10.1111/pbi.13424

**Published:** 2020-06-24

**Authors:** Haifeng Guo, Yawen Zeng, Jilong Li, Xiaoqian Ma, Zhanying Zhang, Qijin Lou, Jin Li, Yunsong Gu, Hongliang Zhang, Jinjie Li, Zichao Li

**Affiliations:** ^1^ State Key Laboratory of Agrobiotechnology/Beijing Key Laboratory of Crop Genetic Improvement College of Agronomy and Biotechnology China Agricultural University Beijing China; ^2^ Biotechnology and Genetic Resources Institute Yunnan Academy of Agricultural Sciences Kunming China; ^3^ State Key Laboratory of Systematic and Evolutionary Botany Institute of Botany Chinese Academy of Sciences Beijing China

**Keywords:** adaptive differentiation, cold tolerance, GWAS, breeding

## Abstract

Genetic studies on cold tolerance at the reproductive stage in rice could lead to significant reductions in yield losses. However, knowledge about the genetic basis and adaptive differentiation, as well as the evolution and utilization of the underlying natural alleles, remains limited. Here, 580 rice accessions in two association panels were used to perform genome‐wide association study, and 156 loci associated with cold tolerance at the reproductive stage were identified. *Os01g0923600* and *Os01g0923800* were identified as promising candidate genes in *qCTB1t*, a major associated locus. Through population genetic analyses, 22 and 29 divergent regions controlling cold adaptive differentiation inter‐subspecies (*Xian*/*Indica* and *Geng*/*Japonica*) and intra‐*Geng,* respectively, were identified. Joint analyses of four cloned cold‐tolerance genes showed that they had different origins and utilizations under various climatic conditions. *bZIP73* and *OsAPX1* differentiating inter‐subspecies evolved directly from wild rice, whereas the novel mutations *CTB4a* and *Ctb1* arose in *Geng* during adaptation to colder climates. The cold‐tolerant *Geng* accessions have undergone stronger selection under colder climate conditions than other accessions during the domestication and breeding processes. Additive effects of dominant allelic variants of four identified genes have been important in adaptation to cold in modern rice varieties. Therefore, this study provides valuable information for further gene discovery and pyramiding breeding to improve cold tolerance at the reproductive stage in rice.

## Introduction

Rice (*Oryza sativa* L.), a staple cereal crop feeding half of the world population, evolved in tropical and subtropical areas and is sensitive to cold stress (Fairhurst and Dobermann, 2002; Huang *et al*., 2012; Zhang *et al*., 2014). Cold damage affects both the vegetative (germination and seedling) and reproductive (booting and flowering) stages (Da Cruz *et al*., 2013). Cold sensitivity at the reproductive stage causes pollen abortion and the consequent sterility, leading to losses in yield (Shinada *et al*., 2013). To meet the growing demand for rice, it is necessary to expand cultivation areas to high‐latitude and high‐altitude regions, where the cold stress occurs more frequently. Estimates of 3‐5 million tons of rice are lost annually in China due to low temperatures in autumn (Zhu *et al*., 2015). Therefore, cold stress at the reproductive stage is a major limiting factor for rice production, especially in high‐latitude and high‐altitude regions. Asian cultivated rice comprises two main subgroups, *Xian*/*Indica* and *Geng*/*Japonica* (Glaszmann, 1987; Wang *et al*., 2018a), which have developed distinctive types of cold adaptability during domestication (Garris *et al*., 2005; Kovach *et al*., 2007). Generally, *Geng* accessions have better cold tolerance than *Xian* (Ma *et al*., 2015). Different climatic conditions in their cultivation areas, mainly temperature, promoted *Xian*‐*Geng* differentiation (Sang and Ge, 2007).

Dissection of the genetic basis of natural variation in cold tolerance is the way to improve low temperature tolerance. Cold tolerance at the reproductive stage is a complex quantitative trait controlled by multiple loci. Through traditional linkage analysis, some QTLs conferring cold tolerance at the reproductive stage have been identified (Andaya and Mackill, 2003; Dai *et al*., 2004; Endo *et al*., 2016; Liang *et al*., 2018; Suh *et al*., 2010; Xu *et al*., 2008; Ye *et al*., 2010; Zeng *et al*., 2009), and a few have been further fine‐mapped (Kuroki *et al*., 2007; Li *et al*., 2018; Shirasawa *et al*., 2012; Zhou *et al*., 2010), but only two genes, *Ctb1* and *CTB4a*, have been cloned and functionally validated (Saito *et al*., 2010; Zhang *et al*., 2017). Recently, genome‐wide association study (GWAS), a new genetic approach, has been used to clarify genetic structure and discover genes underlying important agronomic traits in rice, including grain size, heading date and plant architecture (Huang *et al*., 2010; Si *et al*., 2016; Yano *et al*., 2019; Yano *et al*., 2016; Yu *et al*., 2017). Some associated genes/QTL, such as *OsSAP16*, *bZIP73* and *qPSR10*, controlling cold tolerance at the vegetative stage, have been identified by GWAS (Liu *et al*., 2018; Wang *et al*.,2018b; Xiao *et al*., 2018), but there were few studies regarding cold tolerance at the reproductive stage (Xiao *et al*., 2018), for which the underlying genetic bases are unclear.

Genes for important traits, that were selected and utilized during the domestication of cultivated rice, have different origins and evolutionary paths. Some of them originated directly from wild rice, whereas others were novel mutations selected in cultivated rice. During domestication and adaptation to cold, favourable alleles of *COLD1*, *bZIP73* and *qPSR10*, controlling cold tolerance at the seedling stage, were directly selected from the existing variation in wild rice and promoted the cold adaptation and differentiation of *Geng* as a subspecies (Liu *et al*., 2018; Ma *et al*., 2015; Xiao *et al*., 2018). Contrarily, the favourable alleles of *HAN1* and *CTB4a*, regulating cold tolerance at the seedling and booting stages, respectively, were selected from favourable mutations that occurred during domestication and breeding of *Geng* under cold climatic conditions (Mao *et al*., 2019; Zhang *et al*., 2017).

In this study, genetic and phenotypic data for two association panels comprising 580 accessions were used in GWAS to identify the significant loci and candidate genes controlling variation in cold tolerance at the reproductive stage. Population genetic analyses were also carried out to understand the genetic basis of cold adaptive differentiation. Combined analyses were carried out to reveal the evolution and application of the identified genes for cold tolerance at the reproductive stage. Our findings provide important information for further gene discovery and pyramiding breeding of cold tolerance at the reproductive stage in rice.

## Results

### Phenotypic variation and adaptation to cold between and within subspecies

**Figure 1:**
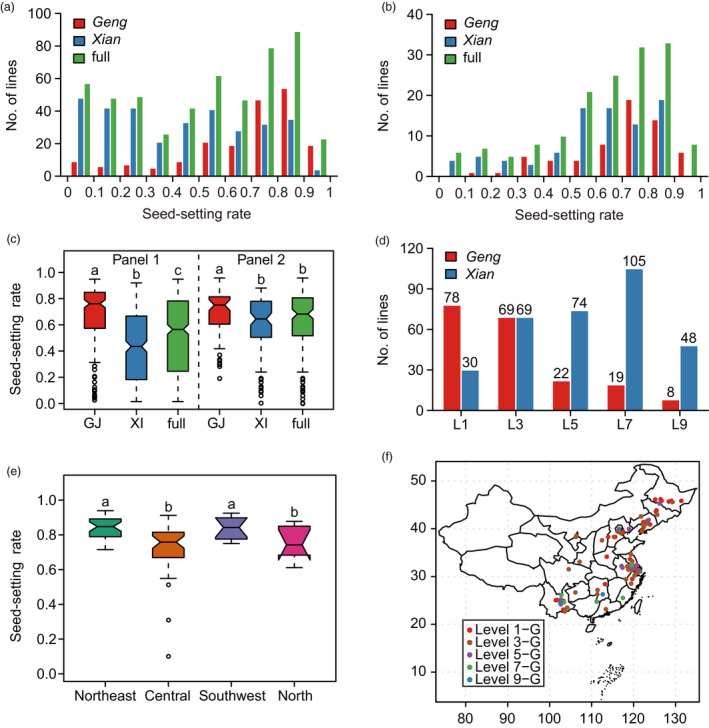
Phenotypic characterization of the association panels. (a, b) Distribution of the seed‐setting rates for accessions in Panel 1 (a) and Panel 2 (b). (c) Comparison of the seed‐setting rates among subgroups in both panels. (d) Distribution of subgroups for different levels of cold tolerance. (e) Comparison of the seed‐setting rates among *Geng* accessions from the southwest plateau, and northeast, central and north China. (f) Distribution of *Geng* accessions from Panel 1 in China. GJ/G, *Geng*; XI/X, *Xian*. Significance of differences was determined by double‐tailed Student’s *t*‐tests.

Five hundred and eighty accessions from 38 countries, including 358 *Xian* and 222 *Geng* accessions (Zhao *et al*., 2018), were phenotyped for this study. These accessions were divided into two panels for different methods of cold treatment. Panel 1 with 522 accessions was treated under natural cold stress conditions in a high‐altitude area (CS‐HAA) (Table S1), where the daily average temperature was below the critical temperature (20°C) required for full fertility (Figure S1a‐c). Panel 2 consisted of 155 accessions that were evaluated under artificial cold stress in deep water (CS‐DW) (Table S2, Figure S1d, e). Phenotypic analysis showed that there was extensive variation in both subspecies in both panels (Table S3, Figure 1a, b). Seed‐setting rates of *Geng* were significantly higher than those of *Xian* in both panels (Figure 1c), indicating that *Geng* had better cold adaptability than *Xian* at the reproductive stage. To screen elite germplasms for genetic improvement of cold tolerance, we classified accessions in Panel 1 into five groups according to seed‐setting rate; 59.8% of cold‐tolerant accessions (levels 1 and 3) were *Geng*, whereas 82.2% of cold‐sensitive accessions (levels 5, 7 and 9) were *Xian* (Figure 1d). Some accessions, mainly from northeast China and the Yungui Plateau (Yunnan and Guizhou provinces) stood out with strong cold tolerance at the reproductive stage (Table S4).

*Geng* cultivars were generally considered to have acquired better cold adaptability than *Xian* during domestication. We confirmed clear differentiation between them in cold adaptability at the reproductive stage (Figure 1c, Figure S2). As the main source of cold‐tolerant germplasms and genes, *Geng* has played important roles in basic research and breeding for cold tolerance. To understand the cold adaptive differentiation within *Geng*, we compared the cold tolerance of *Geng* accessions from different regions in China. Accessions from northeast China and the southwest plateau exhibited stronger cold tolerance than those from north and central China (Figure 1e), indicating that cold adaptability at the reproductive stage also differentiated to some extent within *Geng*.

To verify the underlying external factors affecting adaptive differentiation to cold, the geographic distribution of accessions with different levels of cold tolerance was investigated. The *Xian* subgroup including a large number of cold‐sensitive accessions was mainly distributed in tropical and subtropical areas, whereas *Geng* which included an abundant number of cold‐tolerant accessions had a wider distribution and predominated in temperate regions and subtropical areas with high altitude (Figure 1f, Figure S3). Cold‐sensitive *Geng* accessions were mainly distributed in low‐latitude regions, such as north and central China, whereas cold‐tolerant accessions were mainly distributed in high‐latitude and high‐altitude regions, such as northeast China and the southwest plateau (Figure 1f). These results suggested that different climate conditions, mainly temperature, were the driving force promoting the subspecies differentiation and that the cold‐tolerant *Geng* accessions likely evolved during adaptation of *Geng* to colder climates in high‐latitude and high‐altitude regions.

### Genetic structure of natural variation in cold tolerance revealed by GWAS

**Figure 2:**
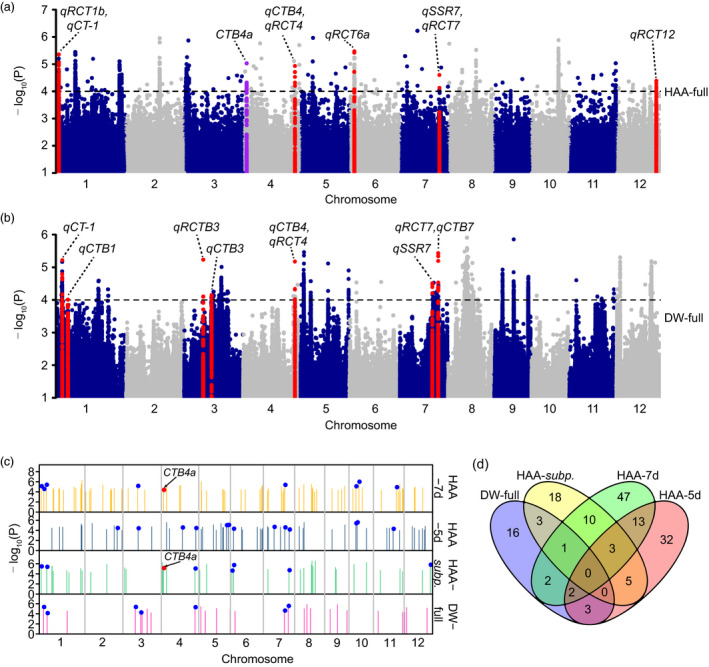
GWAS for cold tolerance at the reproductive stage in association panels. (a, b) Manhattan plots of GWAS in the full population of Panel 1 (a) and Panel 2 (b). Red and purple dots represent associated loci overlapping with reported QTLs and genes, respectively. (c) Summary of the identified loci. Each bar represents an associated locus. Blue and red dots indicate the loci co‐localized with reported QTLs and genes, respectively. (d) Venn diagram showing unique and shared loci identified in different populations. HAA‐7d and HAA‐5d indicate the populations from HAA‐7d‐group 1 to HAA‐7d‐group 4 and from HAA‐5d‐group 5 to HAA‐5d‐group 8, respectively, in Panel 1. HAA‐*subp*. indicates the full population, *Geng* and *Xian* subpopulations in Panel 1. DW‐full indicates the full population in Panel 2.

We performed principal component analysis using SNPs in linkage equilibrium to understand the population structure of the two panels and found that both panels included both *Xian* and *Geng* accessions (Figure S4a, b). This was also supported by neighbour‐joining trees constructed using evenly distributed SNPs (Figure S4c, d). GWAS was performed in two association panels to reveal the genetic basis of natural variation in cold tolerance at the reproductive stage. From the quantile–quantile plots, the compressed mixed linear model was adopted because it better reduced the level of false positives than the general linear model (Figure S5). The significance threshold (*P* < 0.0001) was set based on the permutation tests (Figure S6), and the interval of significant loci was determined according to the population LD decay distance (Figure S7, Table S5). Forty loci associated with cold tolerance at the reproductive stage were identified in Panel 1, including 17, 21 and 10 loci detected in the full population, *Geng* and *Xian* subpopulations, respectively (Figure 2a, Figure S8), and 27 associated loci were identified in the Panel 2 population (Figure 2b).

As different heading dates might affect the evaluation of cold tolerance at the reproductive stage under CS‐HAA conditions, we planted Panel 1 accessions at different dates (Figure S1c); four groups based on seven‐day windows of heading date were separately subjected to GWAS. Seventy‐eight loci associated with cold tolerance at the reproductive stage were detected in Panel 1 using this seven‐day window method (HAA‐7d). Among them, 21, 15, 24 and 22 loci were detected in populations from HAA‐7d‐group 1 to HAA‐7d‐group 4, respectively, and 4 loci were repeatedly detected among different groups (Figure S9). To compare the effect of different grouping windows, we also classified Panel 1 accessions into another four groups based on five‐day windows (HAA‐5d); 58 associated loci were detected, including 12, 9, 19 and 18 loci detected in populations from HAA‐5d‐group 5 to HAA‐5d‐group 8, respectively (Figure S10). There were 18 loci repeatedly detected by both grouping methods (Figure 2c, d), indicating a limited influence of grouping windows on GWAS results.

We identified 156 loci associated with cold tolerance at the reproductive stage in both panels with their detailed information summarized in Tables S6‐S7; they were widely distributed throughout the genome (Figure 2c, Figure S11). Among them, 14 and 8 loci were repeatedly detected between subpopulations and HAA‐7d groups, HAA‐5d groups, respectively, in Panel 1 (Figure 2d). By comparison with QTLs identified by linkage analysis, 27 loci were co‐localized with reported QTLs, and 13 of them were repeatedly detected in association panels (Table S7). Moreover, significant and overlapping signals were repeatedly detected for reported gene *CTB4a* in the HAA‐full and HAA‐7d‐group 3 populations. These results suggested that the grouping method well controlled the influence of heading date under CS‐HAA conditions, and the overlapping QTLs and gene indicated the reliability of our GWAS results.

### Candidate gene analysis of major locus *qCTB1t* and characterization of *CTB4a*

**Figure 3:**
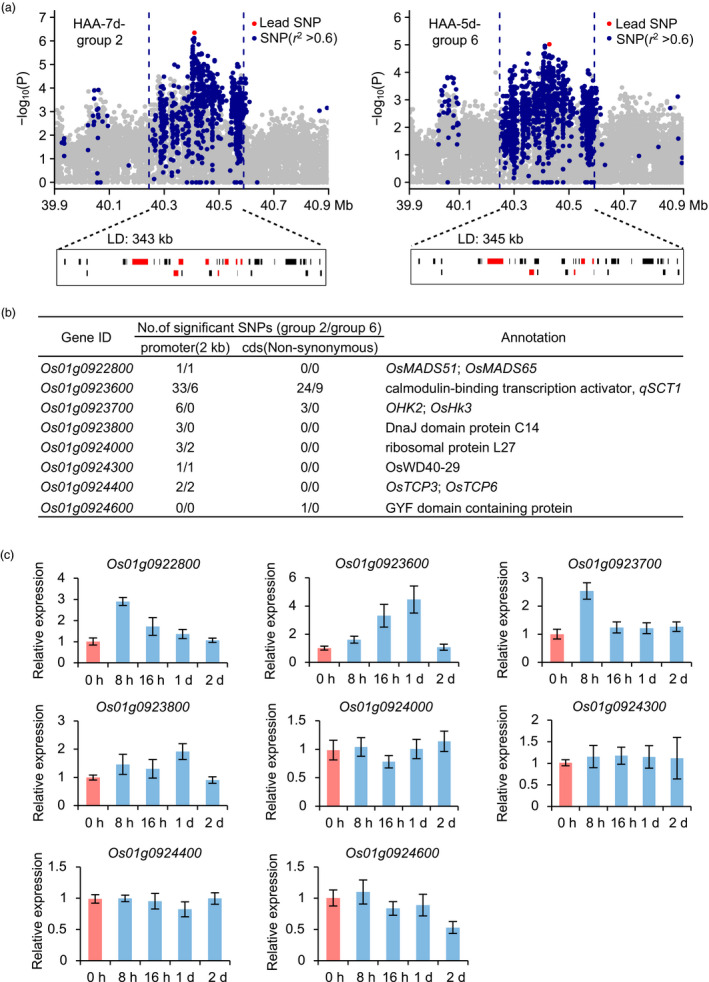
Candidate gene analysis of *qCTB1t*. (a) Local manhattan plots surrounding the peak of *qCTB1t* in HAA‐7d‐group 2 (left) and HAA‐5d‐group 6 (right) populations and corresponding genes overviews. Red bars indicate predicted genes possessing significant SNPs. (b) Details for eight potential candidate genes. (c) Cold‐induced expression analysis of eight potential candidate genes at the reproductive stage. Data represent means ± s.d. (*n* = 3).

Among the identified loci, a novel locus *qCTB1t* at 40–41 Mb on chromosome 1 was repeatedly detected with strong signals (Table S6). To determine the underlying candidate genes, we performed local LD analysis and found that *qCTB1t* corresponded to a 340 kb interval containing 37 predicted genes (Figure 3a). Among them, 8 genes possessing significant SNPs associated with cold tolerance in the HAA‐7d‐group 2 and HAA‐5d‐group 6 populations were selected as potential candidate genes for further analysis (Figure 3b). Tissue expression analysis showed that *Os01g0923600* and *Os01g0923800* were highly expressed in panicles, anthers and pistils, similar to the expression patterns of reported genes regulating cold tolerance at the booting stage (Figure S12). The results of cold‐induced expression analysis showed that *Os01g0922800*, *Os01g0923600*, *Os01g0923700* and *Os01g0923800* were cold‐inducible at the reproductive stage (Figure 3c). In addition, *Os01g0923600*, encoding a calmodulin‐binding transcription activator, was reported to possibly control cold tolerance at the seedling stage (Kim *et al*., 2014). *Os01g0923800*, encoding DnaJ domain protein C14, was annotated as being involved in response to stress (Sarkar *et al*., 2013). We concluded that *Os01g0923600* and *Os01g0923800* were the most likely candidate genes in *qCTB1t*.

Map‐based cloning using a biparental mapping population led to the cloning of *CTB4a*, encoding a receptor‐like kinase. This gene was considered to be important in conferring cold tolerance at the booting stage and enhancing adaptation to cold habitats (Zhang *et al*., 2017). In this study, we repeatedly detected *qCTB4d* on chromosome 4 in the HAA‐full and HAA‐7d‐group 3 populations, which contained *CTB4a* (Figure 2a, Figure S9c). Gene‐based association analysis identified some SNPs in the promoter of *CTB4a* that were significantly associated with cold tolerance at the reproductive stage (Figure S13a). Haplotype analysis showed that there were 7 haplotypes of *CTB4a* in HAA‐full population, and 4 of them were separately distributed in *Geng* or *Xian* subpopulations (Figure S13b). There were significant differences of cold tolerance among different haplotypes (Figure S13b), and accessions containing favourable alleles of *CTB4a* generally exhibited stronger cold tolerance at the reproductive stage (Figure S13c). These results further validated the role of *CTB4a* in regulating the variation in cold tolerance at the reproductive stage.

### Genetic differentiation of cold adaptability inter‐subspecies and intra‐subspecies

**Figure 4:**
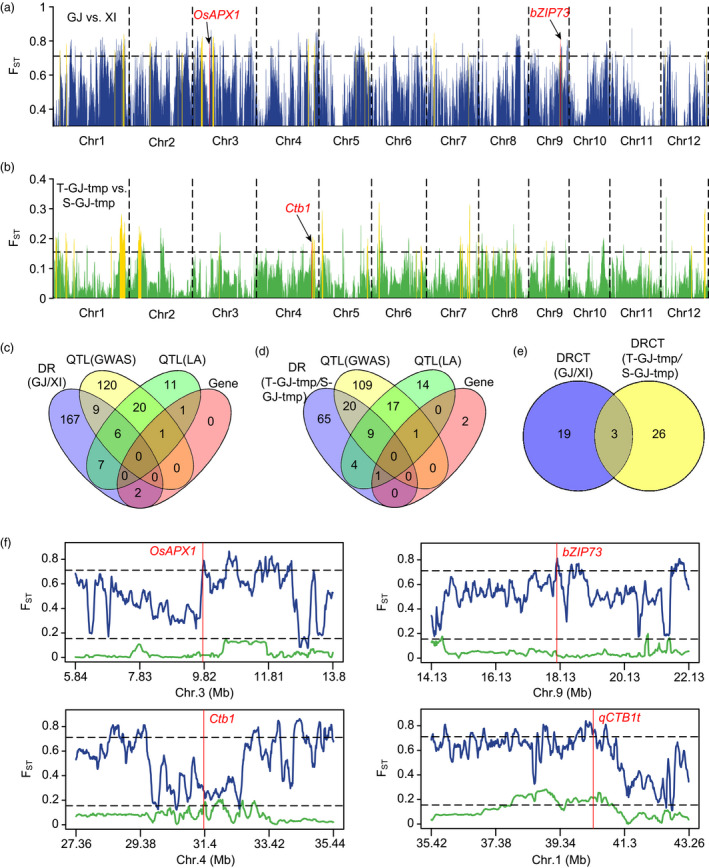
Genomic differentiation of cold adaptability at the reproductive stage. (a, b) Genomic differentiation between *Geng* and *Xian* (a), and within the *Geng* subspecies (b). GJ, *Geng*; XI, *Xian*; T‐GJ‐tmp, cold‐tolerant temperate *Geng*; S‐GJ‐tmp, cold‐sensitive temperate *Geng*. Gold columns indicate the divergent regions overlapping with GWAS signals or reported QTLs. Red columns indicate the divergent regions overlapping with reported cold tolerance genes. Horizontal dashed lines correspond to the top 5% threshold. (c) Comparison of the divergent regions (DR) detected inter‐subspecies with the QTLs/genes identified in this study or reported by linkage analysis. (d) Comparison of the divergent regions (DR) detected intra‐*Geng* with the QTLs/genes identified in this study or reported by linkage analysis. (e) Comparison of the divergent regions related to cold tolerance (DRCT) detected inter‐subspecies and intra‐*Geng*. (f) Genetic differentiation of several reported genes and a major associated locus. Blue and green lines represent the *F*_ST_ between *Geng* and *Xian*, and within *Geng* subspecies, respectively. Horizontal dashed lines correspond to the respective top 5% thresholds. Red vertical lines correspond to the positions of reported genes or associated loci.

To clarify the genetic basis underlying differences in cold adaptability between subspecies at the reproductive stage, we selected 140 *Geng* accessions with the highest cold tolerance level and 169 *Xian* accessions with the most sensitivity to cold, and with wide geographic distributions from Panel 1 (Table S1, Figure S14), and analysed the genetic differentiation between these two subspecies by population differentiation statistics (*F*_ST_). The subspecies were highly divergent in 191 genomic regions (Figure 4a, Table S8). By comparing with loci identified in this study and reported QTLs/genes, we identified 22 highly divergent regions associated with cold tolerance at the reproductive stage. They contained 15 identified loci, 13 reported QTLs and 2 cloned genes (Figure 4c, Table S9). These divergent regions related to cold tolerance (DRCT) covered 1.78% (6.65 Mb) of the reference genome. Two cloned genes, *OsAPX1* and *bZIP73*, conferring cold tolerance at the booting stage (Liu *et al*., 2019; Sato *et al*., 2011), as well as *qCTB1t* identified in this study, showed high divergence between *Xian* and *Geng* (Figure 4a, f). To examine whether these DRCT were under selection, their nucleotide diversity and Tajima’s *D* value were investigated. Based on the estimates of nucleotide diversity ratio, *Xian* generally possessed higher genetic diversity than *Geng* (Mean *π*_*Jap*_/*π*_*Ind*_ = 0.654). In addition, 68% of the DRCT had lower genetic diversity in *Geng* (Figure S15a, Table S9), and the average nucleotide diversity of DRCT in *Geng* (π = 0.00084) was significantly lower than that in *Xian* (*π* = 0.00118; Figure S15c), indicating that these DRCT might be under stronger selection in *Geng* than in *Xian*. Tajima’s *D* analysis showed that about 77% of the DRCT were under directional selection in *Geng*, and most of the DRCT did not escape from neutral evolution in *Xian* (Table S9), indicating that the DRCT between two subspecies were mainly under directional selection in *Geng* during the domestication.

Adaptive differentiation to cold was also analysed intra‐subspecies. Most *Xian* accessions were cold‐sensitive and differentiation of cold adaptability was very weak. However, *Geng* accessions were very different in cold tolerance compared with *Xian*. To investigate the genetic basis of the cold adaptive differentiation within *Geng*, we selected 35 cold‐tolerant and 31 cold‐sensitive Panel 1 accessions from different geographic locations (Table S1, Figure S16) and analysed the population differentiation between them. Ninety‐nine genomic regions were highly divergent between cold‐tolerant and cold‐sensitive *Geng* accessions (Figure 4b, Table S10). Twenty‐nine divergent regions overlapped with 29 identified loci, 13 reported QTLs and 1 cloned gene (Figure 4d, Table S11), covering 3.58% (13.37 Mb) of the reference genome. The cloned cold tolerance gene *Ctb1*, as well as *qCTB1t*, was obviously divergent within *Geng* (Figure 4b, f). Nucleotide diversity analysis showed that these DRCT possessed lower genetic diversity in cold‐tolerant *Geng* accessions (Figure S15b, Table S11). The average nucleotide diversity of DRCT in cold‐tolerant *Geng* (*π* = 0.00053) was significantly lower than that in cold‐sensitive *Geng* (*π* = 0.00252; Figure S15d), indicating that these DRCT could be under stronger selection in cold‐tolerant *Geng* than in cold‐sensitive *Geng*. Tajima’s *D* analysis showed that about 79% of the DRCT displayed directional selection in cold‐tolerant *Geng*, and 55% of the DRCT exhibited balancing selection in cold‐sensitive *Geng* (Table S11), indicating that the DRCT within *Geng* were mainly under directional selection in cold‐tolerant *Geng*, and half of them were under balancing selection in cold‐sensitive *Geng*.

A comparative analysis indicated that 11.5% of the divergent regions between *Xian* and *Geng*, and 29.3% of the divergent regions within *Geng*, contained at least one QTL/gene conferring cold tolerance at the reproductive stage (Figure 4c, d), but only three overlaps were found between the DRCT inter‐subspecies and intra‐*Geng* (Figure 4e). This indicated that there was a higher proportion of DRCT intra‐*Geng* than inter‐subspecies, and the genetic basis of cold adaptation intra‐*Geng* was distinctive from that inter‐subspecies.

### Evolutionary histories and breeding applications of four cold‐tolerance genes

**Figure 5:**
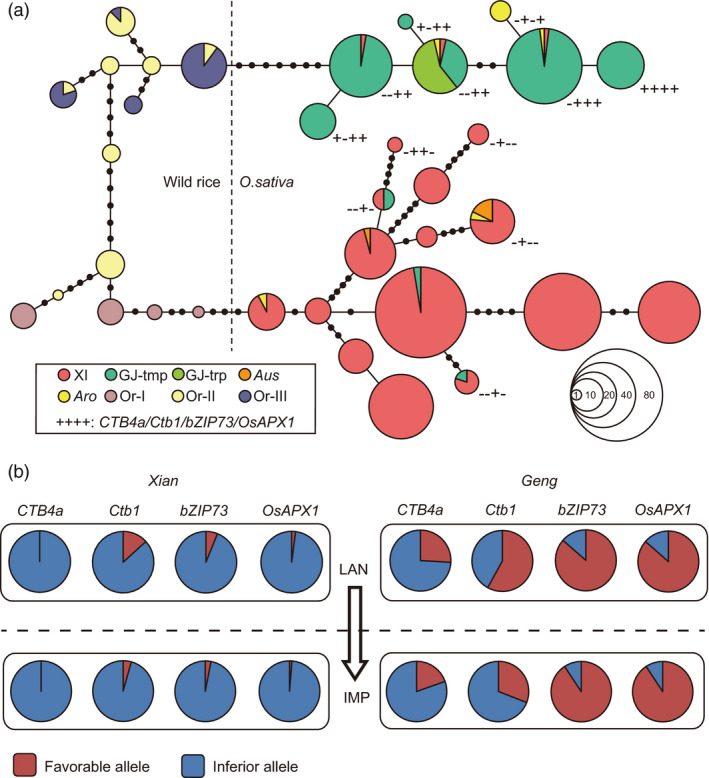
Evolutionary relationship and breeding utilization of four identified genes conferring cold tolerance at the reproductive stage. (a) Evolution relationship of *CTB4a*, *Ctb1*, *bZIP73* and *OsAPX1* revealed by a combined minimum spanning tree. ‘+’ and ‘‐’ represent favourable and inferior alleles, respectively. (b) Allelic changes in *CTB4a*, *Ctb1*, *bZIP73* and *OsAPX1* during rice breeding. XI, *Xian*; GJ‐tmp, temperate *Geng*; GJ‐trp, tropical *Geng*; Or‐I to Or‐III, *O. rufipogon* I to III.

Few genes have been reported to confer cold tolerance at the reproductive stage, and their evolutionary relationships and breeding potential remain unclear. Currently, only four genes, *Ctb1, OsAPX1, CTB4a* and *bZIP73*, have been cloned to regulate cold tolerance at the reproductive stage (Liu *et al*., 2018; Liu *et al*., 2019; Saito *et al*., 2010; Sato *et al*., 2011; Zhang *et al*., 2017). And we also identified them in this study through association analysis and population genetic analysis (Figure 4f, Figure S13). Moreover, we validated their involvement in the regulation of cold tolerance at the reproductive stage through haplotype‐level association analysis (Figure S17a). Among them, *CTB4a* was reported to have 9 haplotypes with its favourable haplotype present in the temperate *Geng* (Zhang *et al*., 2017). And the functional variation site of *bZIP73* was reported divergent between *Geng* and *Xian* (Liu *et al*., 2018). We performed haplotype analysis of *Ctb1* and *OsAPX1* in order to understand their evolution relationships. Three and 6 haplotypes were identified for *Ctb1* in *Geng* and *Xian* subpopulations, respectively, and Hap1 showing the strongest cold tolerance at the reproductive stage was identified as the favourable haplotype of *Ctb1* (Figure S17b, d). There were 2 and 6 haplotypes of *OsAPX1* in *Geng* and *Xian* subpopulations, respectively, and Hap1 of *OsAPX1* was identified as the favourable haplotype (Figure S17c, e). Through a combined minimum spanning tree based on 75 wild rice and 494 cultivated rice accessions (Table S12), we found that the favourable alleles of *bZIP73* and *OsAPX1* evolved directly from the *O. rufipogon* III and were mainly retained in *Geng*. There were a few introgressions from *Geng* to *Xian* for *Ctb1* and *bZIP73* (Figure 5a). The favourable alleles of *CTB4a* and *Ctb1* arose in temperate *Geng* and were subsequently retained (Figure 5a).

To understand the breeding utilization of *CTB4a*, *bZIP73*, *OsAPX1* and *Ctb1*, we investigated the changes of allelic frequencies during rice breeding in 505 cultivated rice accessions (Table S12). The favourable alleles of these four genes were mainly distributed in *Geng,* including landraces and improved varieties, and were rare in *Xian* (Figure 5b). The favourable alleles of *bZIP73* and *OsAPX1* were retained in *Geng* during the domestication and had high proportions in landraces (86% and 88%) and improved varieties (91% and 93%; Figure 5b). On the contrary, there were relatively low proportions of favourable *CTB4a* and *Ctb1* alleles in landraces (26% and 58%) and improved varieties (20% and 31%) of *Geng* (Figure 5b). These results suggested that *bZIP73* and *OsAPX1* have been widely used for improving cold tolerance in *Geng* subspecies and that there is still potential for utilization of favourable *CTB4a* and *Ctb1* alleles in improving cold tolerance at the reproductive stage. To obtain detailed information about the regions of utilization, we analysed the geographic distributions of the four genes. Favourable alleles of *CTB4a* and *Ctb1* were mainly present in high‐latitude and high‐altitude areas (Figure S18), whereas favourable alleles of *bZIP73* and *OsAPX1* were more widely distributed across all *Geng* planting regions (Figure S19), indicating that the utilization of *CTB4a* and *Ctb1* might have promoted the expansion of *Geng* to high‐altitude and high‐latitude areas.

**Figure 6:**
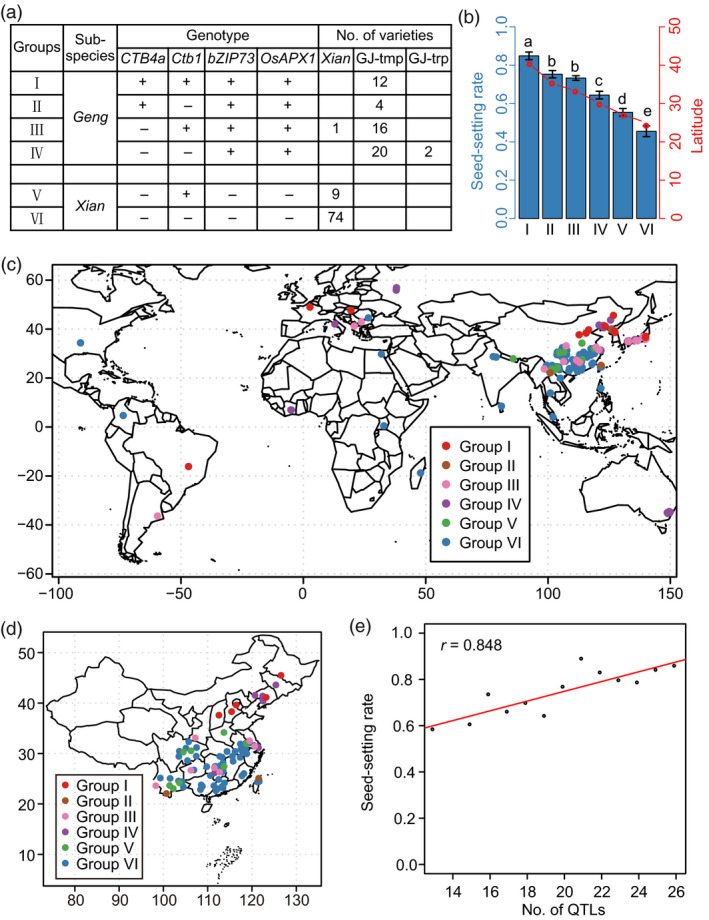
Potential for breeding utilization of genes and loci regulating cold tolerance at the reproductive stage. (a) Combined haplotypes of *CTB4a*, *Ctb1*, *bZIP73* and *OsAPX1*. ‘+’ and ‘−’ represent favourable and inferior alleles, respectively. GJ‐tmp, temperate *Geng*; GJ‐trp, tropical *Geng*. (b) Comparison of the seed‐setting rates and latitudes among different haplotype combinations. (c, d) Distribution of haplotype combinations in worldwide (c) and China (d). (e) Correlation analysis of cold tolerance at the reproductive stage and number of cold tolerance loci.

To clarify the potential for breeding utilization of cold‐tolerance alleles, a combined haplotype analysis for the four genes was performed. Favourable haplotype combinations were present mainly in *Geng* (Figure 6a). Moreover, genotypes combining more favourable alleles showed stronger cold tolerance (Figure 6a, b), implying that pyramiding of favourable alleles would be an effective method to further improve cold tolerance at the reproductive stage. From the geographic distribution, the inferior groups (V and VI) were mainly present in low‐latitude areas, whereas the favourable groups (I to IV) were mainly distributed in the high‐latitude and high‐altitude areas of northeast China, north China and the southwest plateau (Figure 6c, d). In addition, germplasms pyramiding more cold tolerance loci showed stronger cold tolerance at the reproductive stage on the basis of *CTB4a*, *Ctb1*, *bZIP73* and *OsAPX1* (Table S13, Figure 6e). These results indicated that favourable cold tolerance alleles or loci were required to be utilized simultaneously to adapt to the colder climates in high‐latitude and high‐altitude regions.

## Discussion

Cold tolerance is a complex trait affected by environment and artificial cultivated factors. Different growth state and treatment conditions influence the accuracy of cold tolerance evaluation. Due to the influence of heading date and limits to cold treatment facilities, evaluation at the reproductive stage is more difficult than at other growth stages (Zhang *et al*., 2017). Natural low‐temperature conditions and artificial cold water treatment are the most commonly used methods to evaluate cold tolerance at the reproductive stage (Kuroki *et al*., 2007; Suh *et al*., 2010; Zeng *et al*., 2009). In this study, we used both methods to phenotype two association panels. Accessions in Panel 1 were evaluated under natural cold stress conditions. For this, we divided the accessions into different groups based on heading date. In this way, the accessions in each group showed little difference in heading date. The GWAS identified 156 loci associated with cold tolerance at the reproductive stage (Table S6), and some loci were repeatedly detected or were co‐localized with reported QTLs (Table S7). These can be foci for further research.

During the domestication of cultivated rice, cold adaptability differentiated gradually to adapt to the changing ecological habitats. Cold climates in rice‐growing areas promoted *Xian‐Geng* differentiation (Kovach *et al*., 2007). *Geng* cultivars are generally more cold tolerant than *Xian* cultivars (Ma *et al*., 2015). Within subspecies, there also existed germplasms with distinctive cold tolerance, especially in *Geng*, and these germplasms were chosen to construct biparental mapping populations (Dai *et al*., 2004; Zeng *et al*., 2009). We found that cold adaptability differentiated not only inter‐subspecies but also intra*‐Geng* (Figure 1C, E). Cold‐tolerant *Geng* accessions were mainly distributed in regions with relatively lower temperatures, including both temperate areas and subtropical areas with high altitudes (Figure 1f, Figure S3). We propose that temperature difference in different ecological habitats was the main driving force promoting *Xian*‐*Geng* differentiation. On the other hand, the high‐latitude and high‐altitude regions might be the major sources of cold‐tolerant *Geng* accessions. Twenty‐two and 29 DRCT were identified to control cold adaptive differentiation inter‐subspecies and intra‐*Geng*, respectively (Table S9, Table S11), with only a few overlaps being found, thus indicating the distinctive genetic bases of adaptation to cold between them.

We demonstrated that *bZIP73* and *OsAPX1* first evolved from the *O. rufipogon* III and were mainly retained in *Geng*. Subsequently, *CTB4a* and *Ctb1* were retained during the adaptation of temperate *Geng* to colder climatic conditions (Figure 5a). Similar results were found for the genes conferring cold tolerance at the seedling stage. *COLD1*, *bZIP73* and *qPSR10* evolved from wild rice and also promoted the cold adaptation of *Geng* (Liu *et al*., 2018; Ma *et al*., 2015; Xiao *et al*., 2018). *HAN1* was selected from favourable mutations in *Geng* under cold climatic conditions (Mao *et al*., 2019). Therefore, we propose that different cold‐tolerance genes with different origins were retained and utilized under different climates during the cold adaptation. Under normal climatic conditions, several cold‐tolerance genes evolved from the existing variation in wild rice and then retained in *Geng* subgroup. Nevertheless, under cold climatic conditions in high‐latitude and high‐altitude areas, novel mutations enabled temperate *Geng* to adapt to the colder climates.

During crop domestication, favourable alleles for important traits were selected and utilized to meet human demands (Sasaki *et al*., 2002; Yamanaka *et al*., 2004; Yan *et al*., 2011). The four currently reported genes, *Ctb1, OsAPX1, CTB4a* and *bZIP73,* could significantly enhance cold tolerance at the reproductive stage (Liu *et al*., 2019; Saito *et al*., 2010; Sato *et al*., 2011; Zhang *et al*., 2017). The favourable *bZIP73* and *OsAPX1* alleles were retained in *Geng* during the domestication and have been widely utilized to improve cold tolerance in *Geng* subspecies (Figure 5b). While the favourable alleles of *CTB4a* and *Ctb1* were mainly distributed in *Geng* accessions from high‐latitude and high‐altitude areas, there is still potential for further utilizing them to improve cold tolerance at the reproductive stage (Figure 5b, Figure S18a, c). Favourable haplotype combinations with more than two favourable alleles exhibited strong tolerance to cold stress and were mainly present in high‐latitude and high‐altitude areas (Figure 6c, d). And the cold‐tolerant germplasms containing *CTB4a*, *Ctb1*, *bZIP73* and *OsAPX1* also contained the favourable haplotypes of most significantly associated loci (Table S13, Figure 6e). *CTB4a* and *Ctb1*, which originated as novel mutations in *Geng* under cold climatic conditions, were utilized in landraces and leading cultivars, such as Gaoliqiu, Dandongludao, Laoguangtou 83 and Yundao1 from North Korea, northeastern China and Yunnan province. Their utilization might have promoted the expansion of *Geng* to high‐altitude and high‐latitude areas (Figure S18). We propose that further pyramiding of favourable cold tolerance alleles or loci will be an effective method to improve cold tolerance at the reproductive stage. For cold tolerance breeding in high‐latitude and high‐altitude areas and for further expansion of rice growing to much colder regions, both the genes coming from wild rice and those that subsequently arose as mutations in *Geng* and selected under cold conditions should be combined.

## Conclusions

We detected 156 associated loci regulating natural variation in cold tolerance at the reproductive stage through GWAS and identified two candidate genes in the associated locus *qCTB1t*. Cold adaptability differentiated not only inter‐subspecies (between *Xian* and *Geng*) but also intra*‐Geng*. Twenty‐two and 29 DRCT controlling cold adaptive differentiation inter‐subspecies and intra‐*Geng* were identified respectively. There were few overlaps of DRCT between inter‐subspecies and intra‐*Geng*, indicating distinctive genetic bases of cold adaptive differentiation between them. Analyses of four identified genes indicated that cold‐tolerance genes with different origins were retained and utilized during the adaptation of rice to colder environments. Our findings confirmed that gene pyramiding will be an effective method to improve cold tolerance at the reproductive stage in rice.

## Experimental procedures

### Plant materials and sequencing

A total of 580 cultivated rice accessions from 38 countries, including 156 accessions from the mini‐core collection (Zhang *et al*., 2011) and 424 accessions from the International Rice Molecular Breeding Network (Yu *et al*., 2003), were phenotyped. Sequencing data were available from the 3000 Rice Genome Project (3KRGP) with an average sequencing depth of 15× (Alexandrov *et al*., 2015; Wang et al., 2018a). For an evolutionary analysis, an additional 123 cultivated rice accessions from 3KRGP and 75 wild rice accessions were added along with sequencing data for 65 wild rice accessions obtained from the published data (Huang *et al*., 2012).

### Phenotyping

In the summer of 2014, 155 accessions in Panel 2 were sown at the ShangZhuang Experimental Station of China Agricultural University in Beijing and transplanted to the field (12.5 cm × 25 cm length plots) 30 days later. Five plants of each accession at the reproductive stage were transferred to a pool irrigated with cold water (16–18°C) for one week and then transplanted back to the field. After harvest, the relative seed‐setting rates of the treated plants were assessed and recorded. In the summer of 2015, 522 accessions in Panel 1 were sown at Yuxi (altitude, 1638 m) in Yunnan province, at 10‐day intervals. Heading dates were recorded, and average seed‐setting rates of five plants for each accession flowering at the same time were investigated after harvest. Detailed descriptions of two cold treatment methods were described in Zhang *et al* (2017).

According to the seed‐setting rates, five levels of cold tolerance at the reproductive stage were set, including level 1 (108 accessions with seed‐setting rates ≥ 80%), level 3 (138 accessions with seed‐setting rates 60%–80%), level 5 (96 accessions with seed‐setting rates 40%–60%), level 7 (124 accessions with seed‐setting rates 10%–40%) and level 9 (56 accessions with seed‐setting rates < 10%).

### Population structure analysis

There were 6 997 165 and 4 625 142 SNPs in total in Panels 1 and 2, respectively. By using PLINK version 1.9 (window 50 bp, step size 5 bp, *r* ^2^ < 0.3; Purcell *et al*., 2007), 316 749 and 117 639 SNPs in linkage equilibrium were screened from Panels 1 and 2, respectively, and were used to perform principal component analysis through GCTA software (Yang *et al*., 2011); 665 057 and 116 903 SNPs evenly distributed throughout the genome were screened from Panels 1 and 2, respectively, using an in‐house perl script and were used to construct neighbour‐joining tree in MEGA version 7 with the bootstrap method and 1000 replicates (Kumar *et al*., 2016).

### GWAS

Totals of 3 851 692 and 3 002 287 high‐quality SNPs (MAF ≥ 5%, missing rate < 25%) from Panels 1 and 2, respectively, were used to perform GWAS using both the general linear model and compressed mixed linear model in the GAPIT package operated in an R environment (Tang *et al*., 2016). To avoid the possible influence of heading date, GWAS for cold tolerance evaluated under CS‐HAA conditions was conducted in multiple populations divided by five and seven days‐to‐heading (DTH) interval groups, including HAA‐7d‐group 1 (168 accessions, DTH from 97 to 103 days), HAA‐7d‐group 2 (167 accessions, DTH from 104 to 110 days), HAA‐7d‐group 3 (133 accessions, DTH from 112 to 118 days), HAA‐7d‐group 4 (110 accessions, DTH from 120 to 126 days), HAA‐5d‐group 5 (98 accessions, DTH from 93 to 97 days), HAA‐5d‐group 6 (130 accessions, DTH from 103 to 107 days), HAA‐5d‐group 7 (122 accessions, DTH from 110 to 114 days) and HAA‐5d‐group 8 (109 accessions, DTH from 118 to 122 days). The genome‐wide significance threshold was determined by permutation tests with 1000 replications (Zhao *et al*., 2018). A region containing more than 3 consecutive significant SNPs was considered a single associated signal, and the SNP with the minimum *P*‐value within the associated signal was considered to be the lead SNP. For the analysis of candidate genes of the associated signal, the continuous region closely linked to the lead SNP (*r* ^2^ ≥ 0.6) was considered as the local LD interval (Yano *et al*., 2016). The significance threshold was determined using 0.05/N, where N represented the total number of SNPs.

### Population genetic analysis

The genome‐wide LD decay of the association populations was determined using PopLDdecay version 3.4 (Zhang *et al*., 2019) with parameters as follows: ‐maxdist 5000 ‐maf 0.05 ‐miss 0.25. The LD decay distance was determined as the LD decays to half of the maximum value.

To clarify the differentiation and selection of cold adaptability at the reproductive stage, several population genetic parameters, including the population differentiation statistics (*F*_ST_), nucleotide diversity (π) and Tajima’s *D*, were calculated using VCFtools software (Danecek *et al*., 2011). *F*_ST_ and nucleotide diversity were computed using 100‐kb windows and 10‐kb steps, and the Tajima’s *D* was calculated using 10‐kb windows. Sliding windows with the top 5% of *F*_ST_ values were identified as divergent windows. Adjacent divergent windows were merged into single divergent regions. Highly divergent regions overlapping with loci identified in this study or reported cold‐tolerance QTLs/genes at the reproductive stage were defined as the divergent regions related to cold tolerance (DRCT) at the reproductive stage. For the identification of DRCT inter‐subspecies, only the loci detected in the full population, but neither in *Xian* nor in *Geng* subpopulations, were identified to contribute to the cold adaptive differentiation inter‐subspecies.

To analyse the selection of divergent regions related to cold tolerance, regions with an average Tajima's *D* < −1 and Tajima's *D *> 1 in corresponding populations were further filtered (Qiu *et al*., 2017; Xia *et al*., 2019; Zhao *et al*., 2018).

### Expression pattern analysis

*Geng* landrace Lijiangxiaoheigu from Yunnan province was transferred to a phytotron (16–17°C) at the reproductive stage, and young panicles were sampled at different time points after cold treatment. Total RNA was extracted using RNAiso Plus (Takara, Japan), and cDNA was generated using an M‐MLV reverse transcriptase (Takara, Japan). qRT‐PCR was performed on an ABI 7500 Real‐time PCR system (Applied Bio‐Systems). *OsActin1* was used as the internal reference. Each experiment was performed with three biological samples and each sample assayed with three technical replications. Data for tissue expression analysis were collected from the Rice Genome Annotation Project website (http://rice.plantbiology.msu.edu/). Primers are listed in Table S14.

### Evolutionary analysis

For evolutionary analysis, haplotypes of 494 cultivated rice and 75 wild rice accessions were divided using DnaSP 5.10 software (Librado and Rozas, 2009). A minimum spanning tree among haplotypes was calculated using Arlequin version 3.5 (Excoffier and Lischer, 2010) and drawn in Hapstar‐0.6 (Teacher and Griffiths, 2011).

## Conflict of interest

The authors declare that they have no competing interests.

## Author contributions

H.G., Jinjie Li and Z.L. designed the research. H.G., Y.Z., Jilong Li and X.M. performed most of experiments. Z.Z., Q.L., Jin Li, Y.G. and H.Z. performed part of the experiments. H.G. and Y.Z. analysed the data. Jinjie Li and Z.L. conceived and supervised the project. H.G., Jinjie Li and Z.L. wrote the manuscript.

## Supporting information


**Figure S1** Evaluation of cold tolerance at the reproductive stage under CS‐HAA and CS‐DW conditions.
**Figure S2** Cold adaptive differentiation between *Xian* and *Geng* and relationship between cold tolerance and latitude.
**Figure S3** Geographic distribution of the accessions in Panel 1
**Figure S4** Population structure of the association panels.
**Figure S5** Quantile‐quantile plots for the general linear model (GLM) and compressed mixed linear model (CMLM) in different association populations.
**Figure S6** Genome‐wide threshold for GWAS based on the permutation tests.
**Figure S7** LD decay of the association populations.
**Figure S8** Loci associated with cold tolerance at the reproductive stage identified in subgroups of Panel 1.
**Figure S9** Loci associated with cold tolerance at the reproductive stage identified in Panel 1 based on a seven‐day grouping interval.
**Figure S10** Loci associated with cold tolerance at the reproductive stage identified in Panel 1 based on a five‐day grouping interval.
**Figure S11** Distribution of 156 loci on rice chromosomes.
**Figure S12** Tissue expression of important predicted genes in *qCTB1t* based on the data from the RGAP website.
**Figure S13** Association analysis of *CTB4a* in HAA‐full population.
**Figure S14** Characterization of 140 *Geng* and 169 *Xian* accessions from Panel 1.
**Figure S15** Nucleotide diversity of DRCT and genomic average in different populations.
**Figure S16** Characterization of 35 cold‐tolerant and 32 cold‐sensitive temperate *Geng* accessions from Panel 1.
**Figure S17** Association and haplotype analyses for cloned genes conferring cold tolerance at the reproductive stage in 132 accessions from Panel 2.
**Figure S18** Allelic distributions of *CTB4a* and *Ctb1* in the world (left) and China (right).
**Figure S19** Allelic distributions of *bZIP73* and *OsAPX1* in the world (left) and China (right).

**Table S1** The 522 accessions in Panel 1 evaluated under natural cold stress in a high‐altitude area.
**Table S2** The 155 accessions in Panel 2 evaluated under artificial cold stress in deep water.
**Table S3** Statistics of the seed‐setting rates of accessions used in this study.
**Table S4** Selected rice germplasms from level 1 with strong cold tolerance at the reproductive stage.
**Table S5** LD decay of different association populations.
**Table S6** Information of the loci associated with cold tolerance at the reproductive stage identified by GWAS in different association populations.
**Table S7** Information of the loci overlapping with QTLs identified by linkage analysis.
**Table S8** Genomic regions differentiated between *Geng* and *Xian*.
**Table S9** Population genetic parameters of DRCT between *Geng* and *Xian*.
**Table S10** Genomic regions differentiated within the *Geng* subspecies.
**Table S11** Population genetic parameters of DRCT within the *Geng* subspecies.
**Table S12** Accessions used in the analyses of evolution and breeding potential.
**Table S13** Pyramiding of loci overlapping with reported cold‐tolerance QTLs at the reproductive stage in temperate *Geng* accessions from Panel 1 which contain *CTB4a*, *Ctb1*, *bZIP73* and *OsAPX1*.
**Table S14** Primers used in the expression pattern analysis.
