# Supplementary material for: Differentiation, evolution and utilization of natural alleles for cold adaptability at the reproductive stage in rice
Source: Plant Biotechnol J. 2020 Jun 24;18(12):2491–503. doi: 10.1111/pbi.13424 (PMC7680545; doi:10.1111/pbi.13424)
Supplement: Supplementary file 1 — Figure S1 Evaluation of cold tolerance at the reproductive stage under CS‐HAA and CS‐DW conditions. Figure S2 Cold adaptive differentiation between Xian and Geng and relationship between cold tolerance and latitude. Figure S3 Geographic distribution of the accessions in Panel 1 Figure S4 Population structure of the association panels. Figure S5 Quantile‐quantile plots for the general linear model (GLM) and compressed mixed linear model (CMLM) in different association populations. Figure S6 Genome‐wide threshold for GWAS based on the permutation tests. Figure S7 LD decay of the association populations. Figure S8 Loci associated with cold tolerance at the reproductive stage identified in subgroups of Panel 1. Figure S9 Loci associated with cold tolerance at the reproductive stage identified in Panel 1 based on a seven‐day grouping interval. Figure S10 Loci associated with cold tolerance at the reproductive stage identified in Panel 1 based on a five‐day grouping interval. Figure S11 Distribution of 156 loci on rice chromosomes. Figure S12 Tissue expression of important predicted genes in qCTB1t based on the data from the RGAP website. Figure S13 Association analysis of CTB4a in HAA‐full population. Figure S14 Characterization of 140 Geng and 169 Xian accessions from Panel 1. Figure S15 Nucleotide diversity of DRCT and genomic average in different populations. Figure S16 Characterization of 35 cold‐tolerant and 32 cold‐sensitive temperate Geng accessions from Panel 1. Figure S17 Association and haplotype analyses for cloned genes conferring cold tolerance at the reproductive stage in 132 accessions from Panel 2. Figure S18 Allelic distributions of CTB4a and Ctb1 in the world (left) and China (right). Figure S19 Allelic distributions of bZIP73 and OsAPX1 in the world (left) and China (right). [file PBI-18-2491-s001.pdf]

Differentiation, evolution and utilization of natural alleles for cold adaptability at the reproductive stage in rice

Haifeng Guo[1,†], Yawen Zeng[2,†], Jilong Li[1,3], Xiaoqian Ma[1], Zhanying Zhang[1], Qijin Lou[1], Jin Li[1], Yunsong Gu[1], Hongliang Zhang[1], Jinjie Li[1,*] and Zichao Li[1,*]

[1] State Key Laboratory of Agrobiotechnology/Beijing Key Laboratory of Crop Genetic Improvement, College of Agronomy and Biotechnology, China Agricultural University, Beijing 100193, China

[2] Biotechnology and Genetic Resources Institute, Yunnan Academy of Agricultural Sciences, Kunming 650205, China

[3] State Key Laboratory of Systematic and Evolutionary Botany, Institute of Botany, Chinese Academy of Sciences, Beijing 100093, China

*Correspondence (emails: lijinjie@cau.edu.cn; lizichao@cau.edu.cn)

[†] These authors contributed equally to this work.

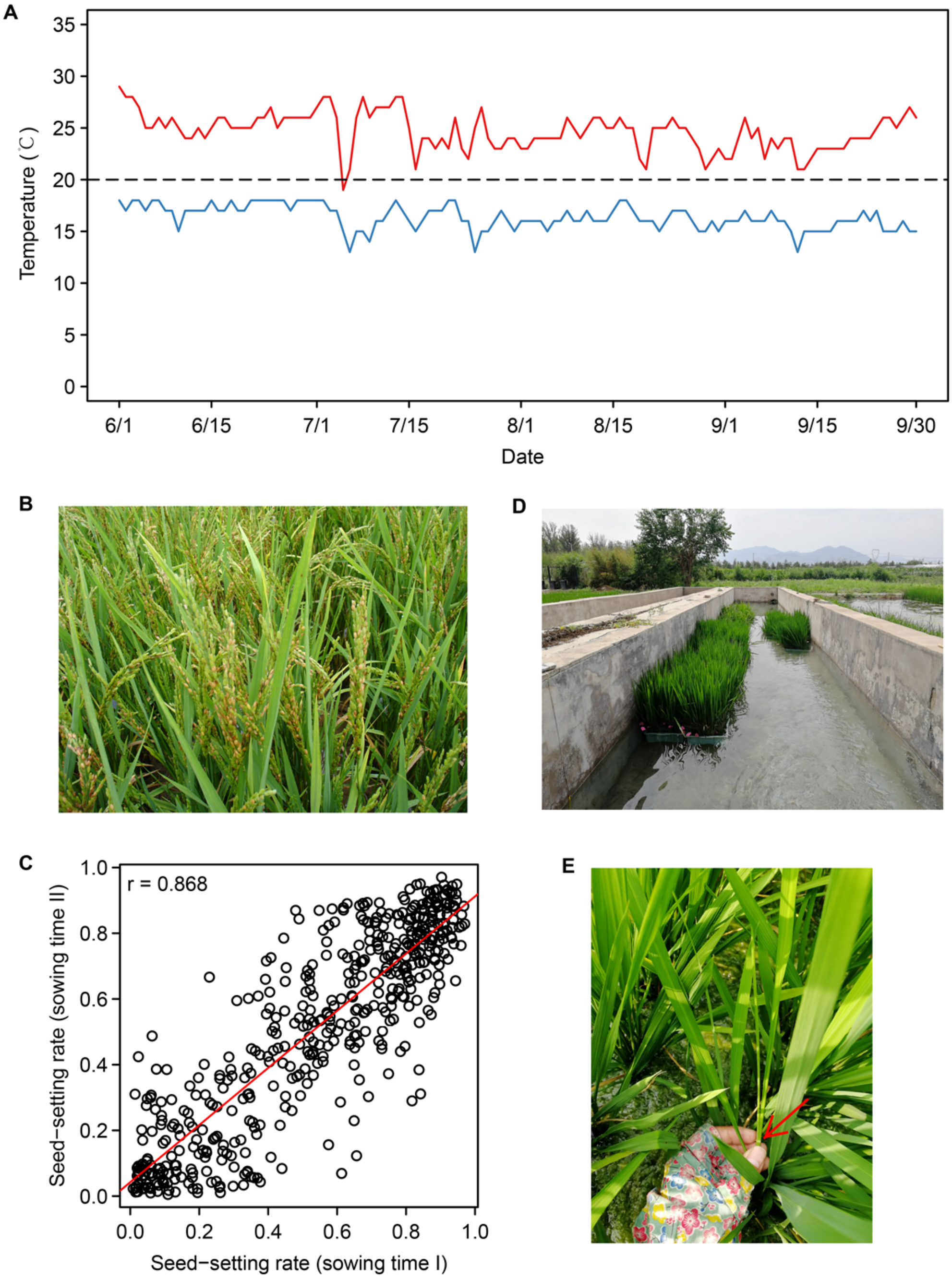

Figure S1. Evaluation of cold tolerance at the reproductive stage under CS-HAA and CS-DW conditions. (A) Temperature records for Yuxi city in summer 2015. The horizontal dotted line represents the critical temperature of cold stress at the reproductive stage. Red and blue lines represent daily maximum and minimum temperatures, respectively. (B) Cold treatment for accessions in Panel 1 under CS-HAA conditions in the field. (C) Correlation analysis of the seed-setting rates for accessions in Panel 1 sown at different times. (D, E) Deep water treatment of accessions in Panel 2. The red arrow in E indicates the stage when the pulvinus of the flag leaf grows to the penultimate leaf level.

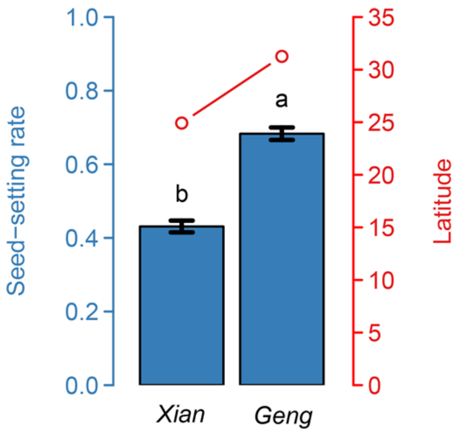

Figure S2. Cold adaptive differentiation between *Xian* and *Geng* and relationship between cold tolerance and latitude.

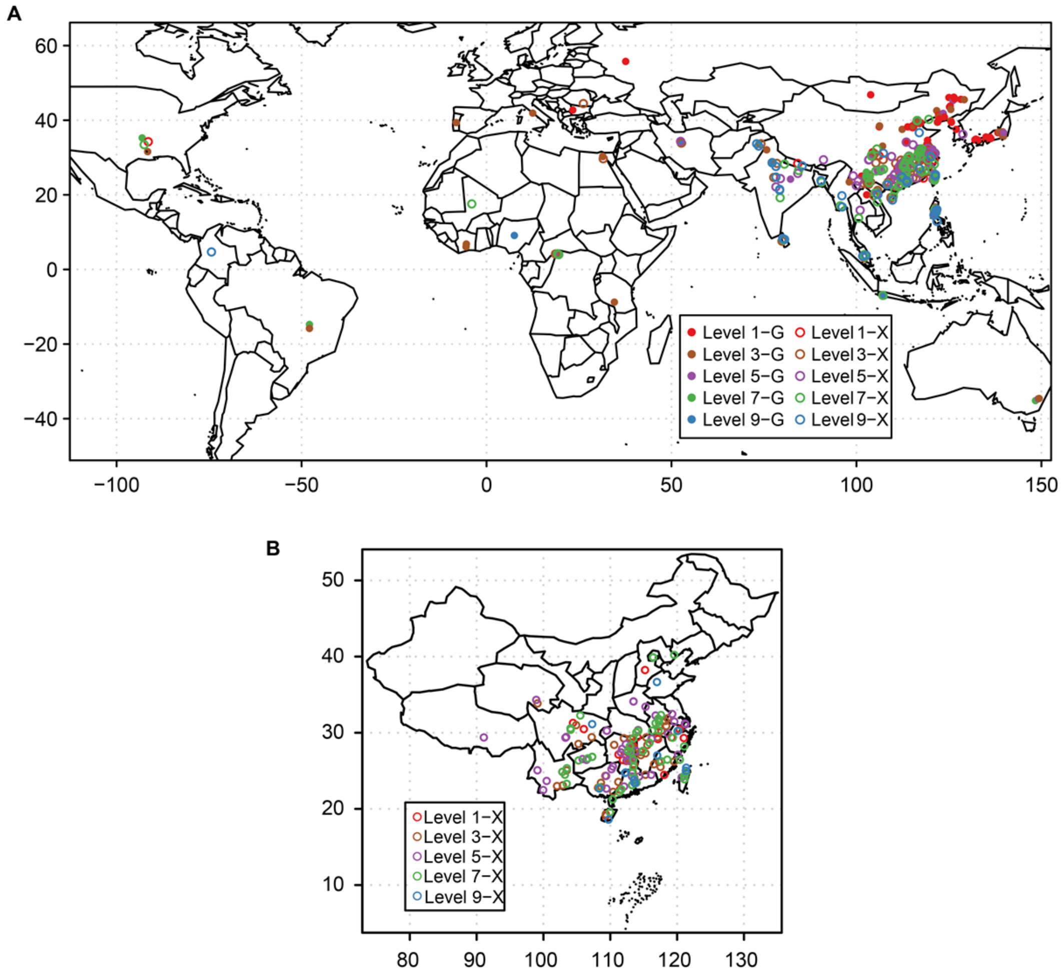

Figure S3. Geographic distribution of the accessions in Panel 1. (A) Worldwide distribution of the accessions. (B) Distribution of *Xian* accessions in China. X, *Xian*; G, *Geng*.

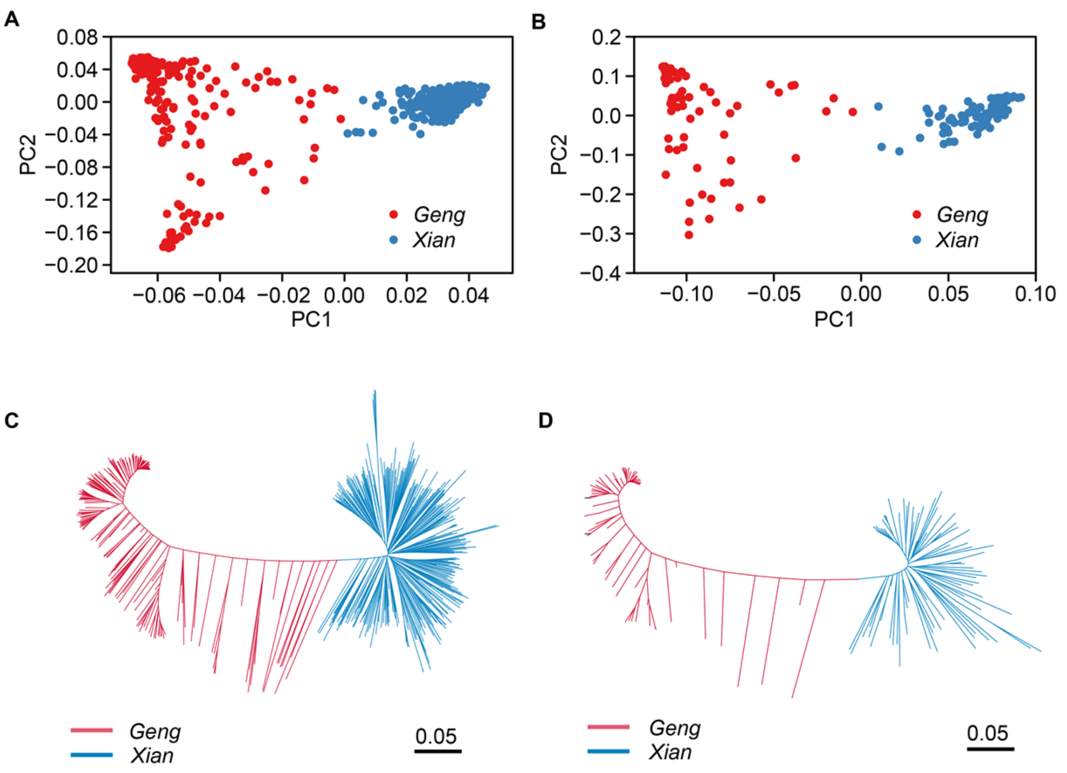

Figure S4. Population structure of the association panels. (A, B) Principal component analyses for accessions in Panel 1 (A) and Panel 2 (B) based on SNPs in linkage equilibrium. (C, D) Neighbor-joining trees of accessions in Panel 1 (C) and Panel 2 (D) constructed using the evenly distributed SNPs.

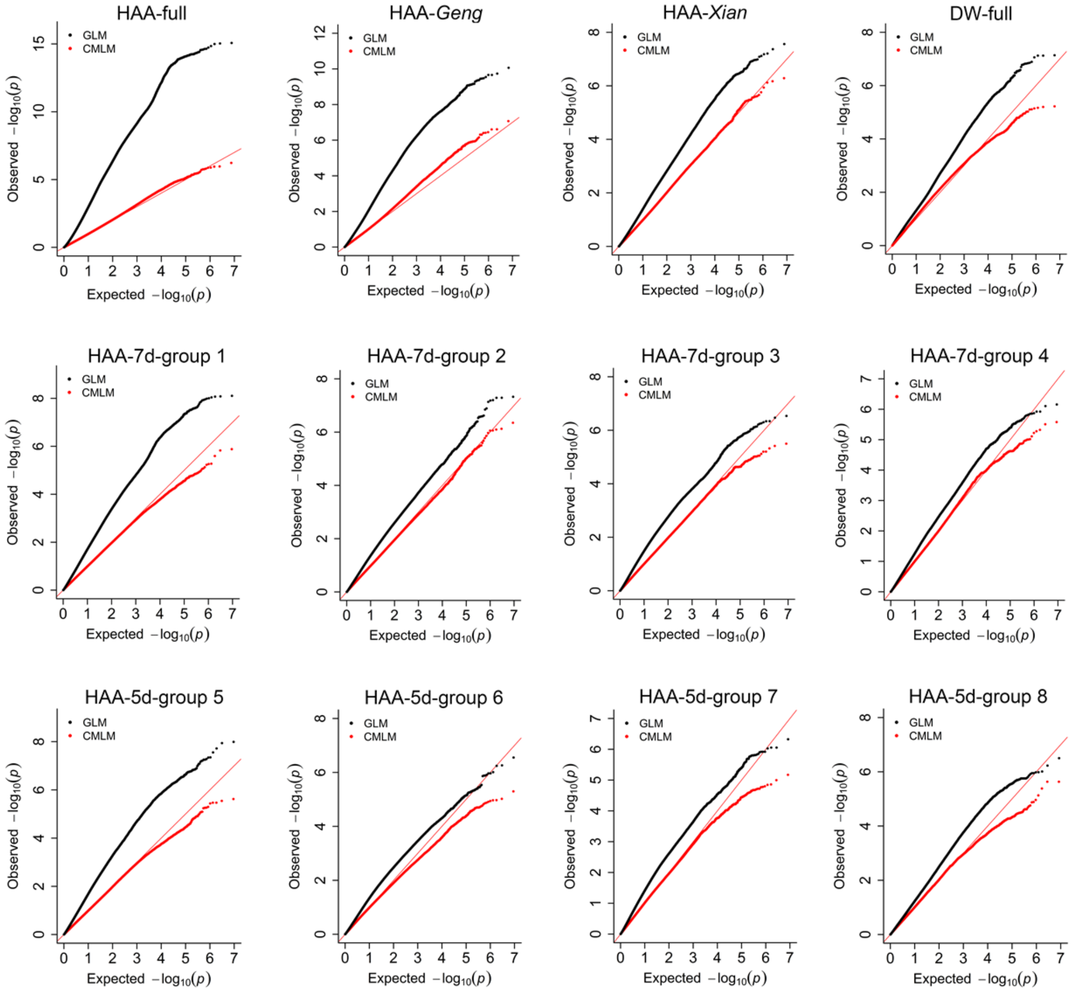

Figure S5. Quantile-quantile plots for the general linear model (GLM) and compressed mixed linear model (CMLM) in different association populations.

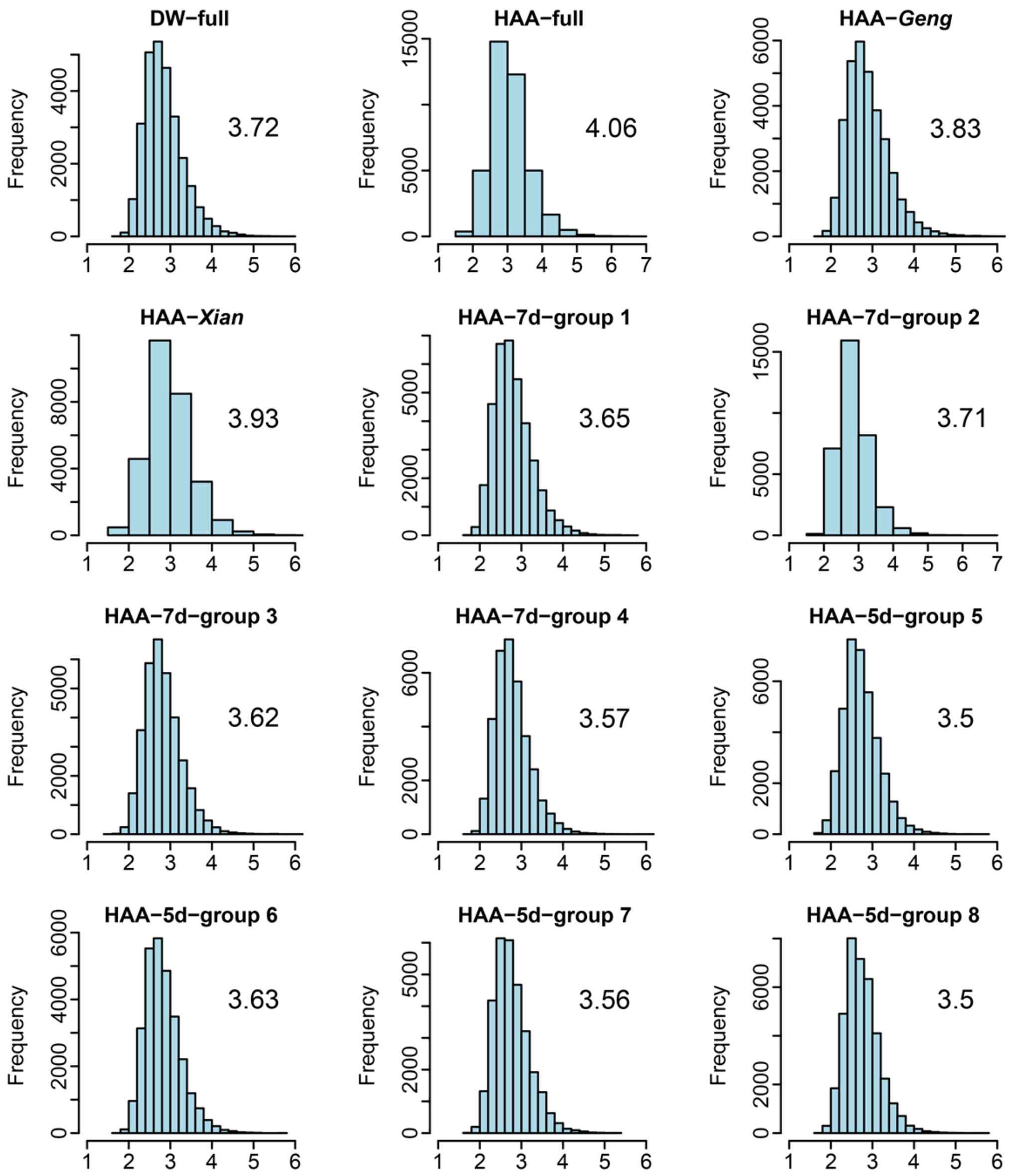

Figure S6. Genome-wide threshold for GWAS based on the permutation tests. The predicted thresholds are given on the right side of each diagram.

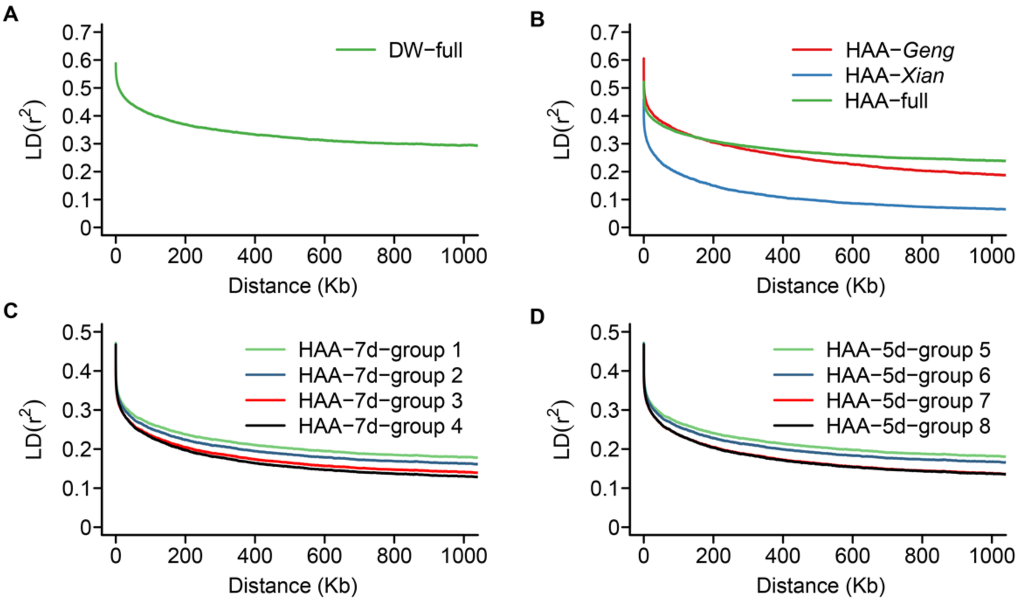

Figure S7. LD decay of the association populations. (A) LD decay of the full population in Panel 2. (B) LD decay of *Geng*, *Xian* and the full population in Panel 1. (C) LD decay of populations from HAA-7d-group 1 to HAA-7d-group 4 in Panel 1. (D) LD decay of populations from HAA-5d-group 5 to HAA-5d-group 8 in Panel 1.

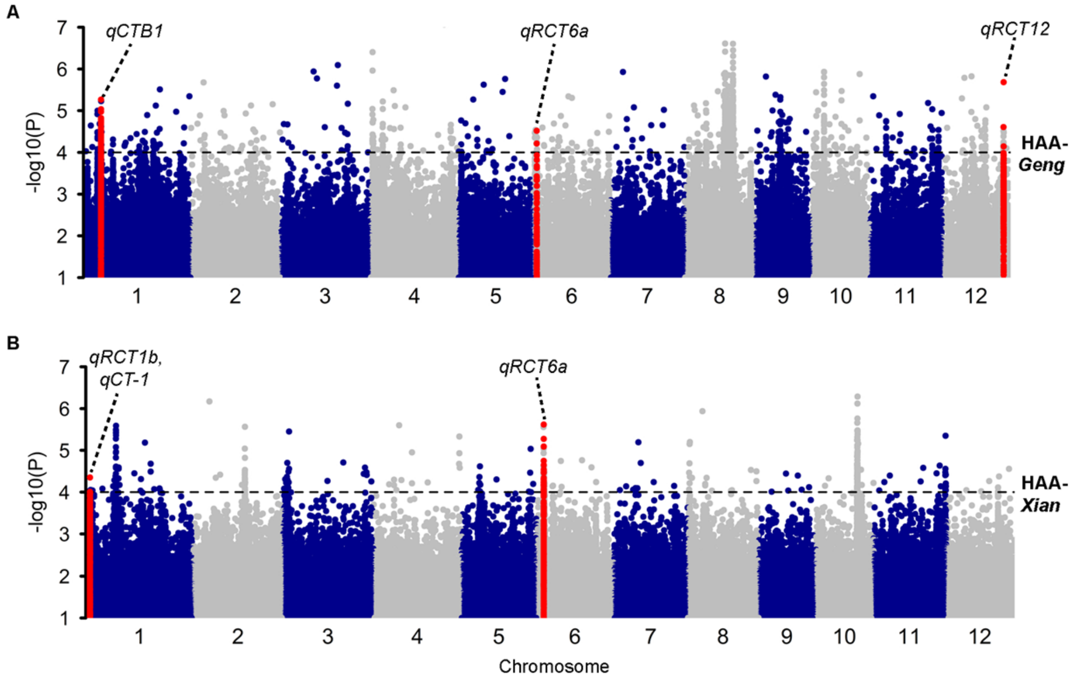

Figure S8. Loci associated with cold tolerance at the reproductive stage identified in subgroups of Panel 1. (A, B) Manhattan plots of GWAS in *Geng* (A) and *Xian* (B) of Panel 1. Red dots represent the associated loci overlapping with reported QTLs.

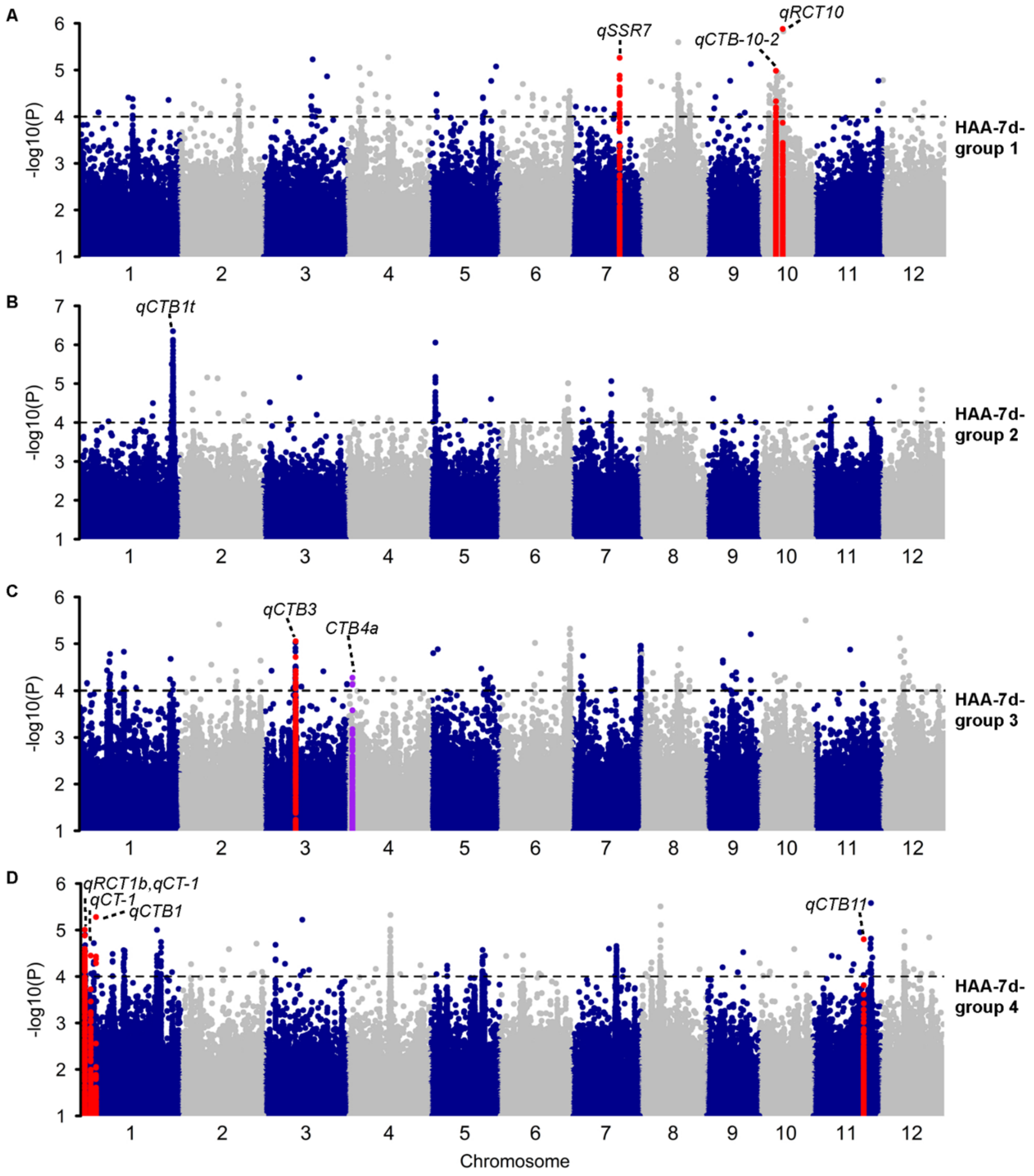

Figure S9. Loci associated with cold tolerance at the reproductive stage identified in Panel 1 based on a seven-day grouping interval. Manhattan plots from the top to the bottom correspond to the GWAS in HAA-7d-group 1 (A), HAA-7d-group 2 (B), HAA-7d-group 3 (C) and HAA-7d-group 4 (D), respectively. Red and purple dots represent the associated loci overlapping with reported QTLs and genes, respectively.

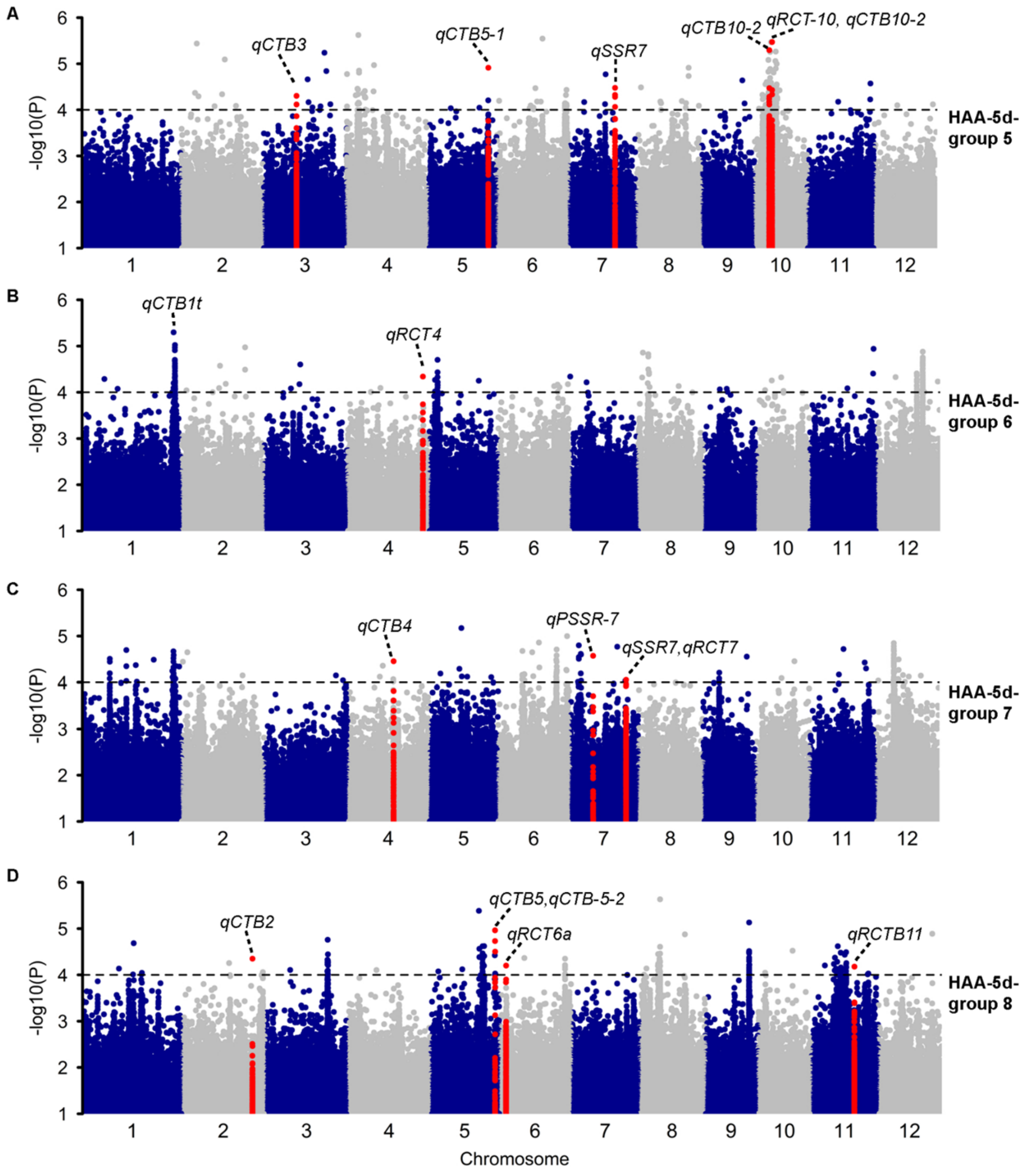

Figure S10. Loci associated with cold tolerance at the reproductive stage identified in Panel 1 based on a five-day grouping interval. Manhattan plots from the top to the bottom correspond to the GWAS in HAA-5d-group 5 (A), HAA-5d-group 6 (B), HAA-5d-group 7 (C) and HAA-5d-group 8 (D), respectively. Red dots represent the associated loci overlapping with reported QTLs.

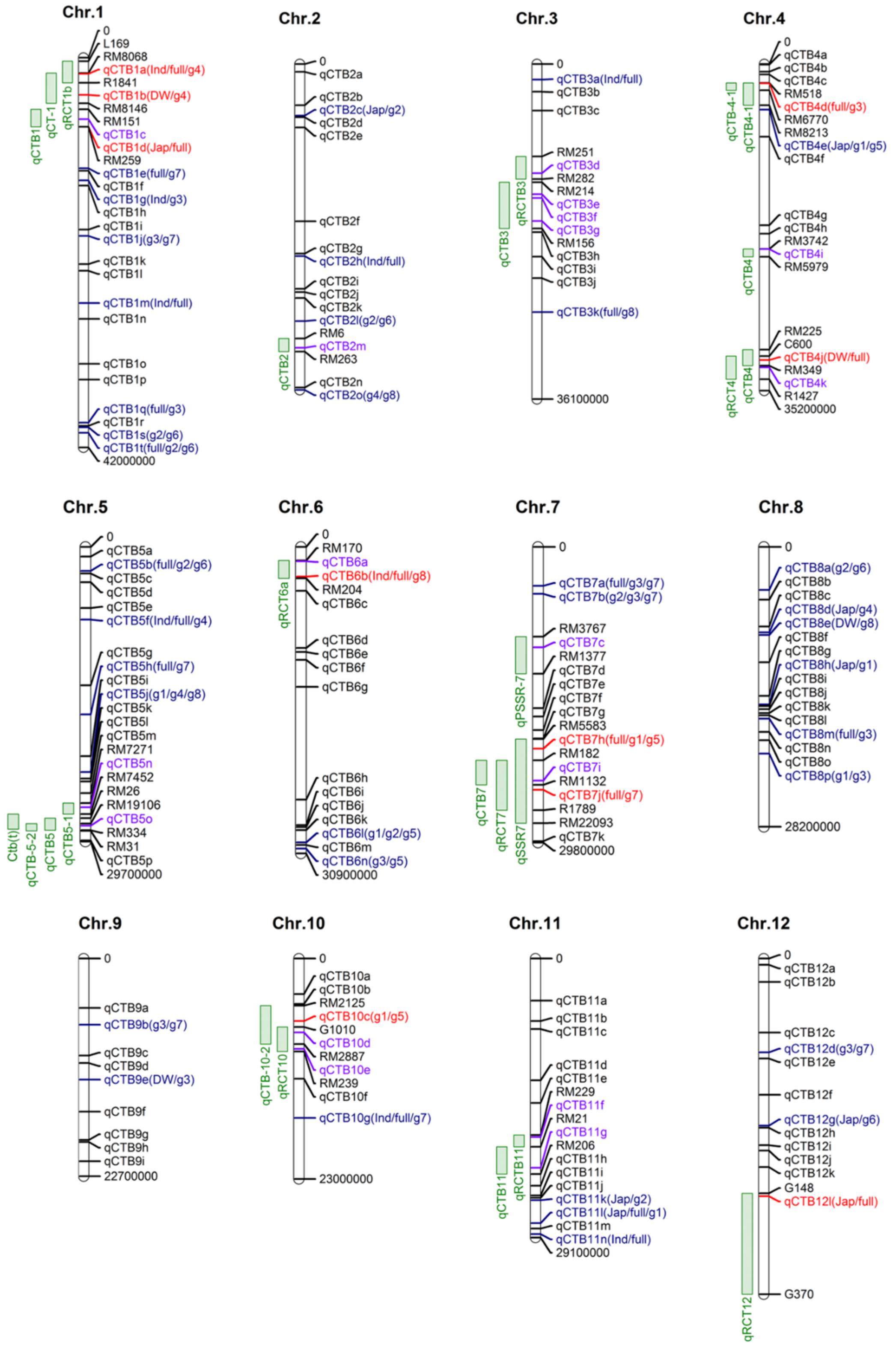

Figure S11. Distribution of 156 loci on rice chromosomes. The associated loci are marked on the right of chromosomes according to their physical positions; the reported overlapping loci are marked on the left with markers shown on the right. The loci in blue represent those repeatedly detected in different populations. Loci in purple represent those previously reported by linkage analysis. Loci in red represent those not only reported by linkage analysis, but also repeatedly detected in this study.

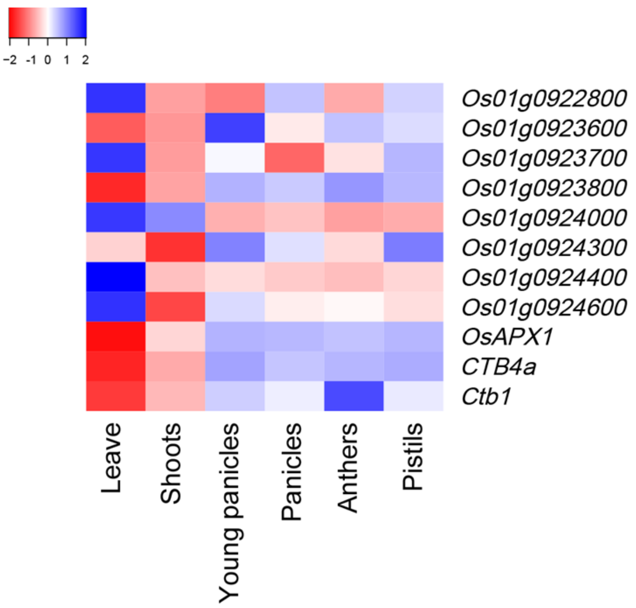

Figure S12. Tissue expression of important predicted genes in *qCTB1t* based on the data from the RGAP website.

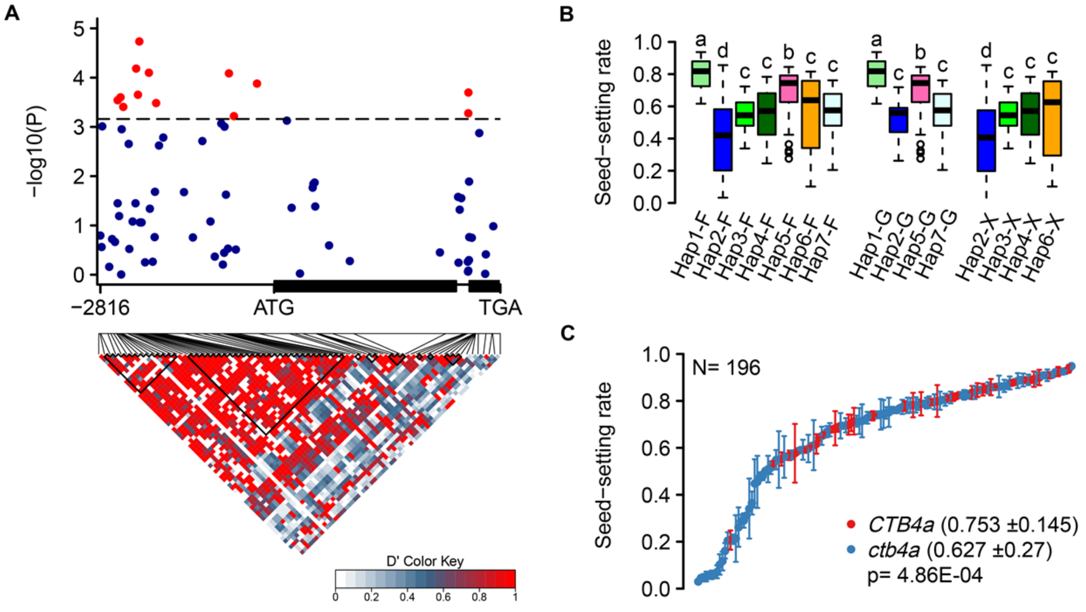

Figure S13. Association analysis of *CTB4a* in HAA-full population. (A) Gene-based association analysis of *CTB4a*. (B) Comparison of the seed-setting rates among different haplotypes of *CTB4a.* Haplotypes with the F, G and X suffixes indicate that the haplotypes were identified in the full population, *Geng* and *Xian* subpopulations respectively. (C) Seed-setting rates of *Geng* accessions from Panel 1 containing different *CTB4a* alleles. Significance of differences were determined by double-tailed Student's *t*-tests.

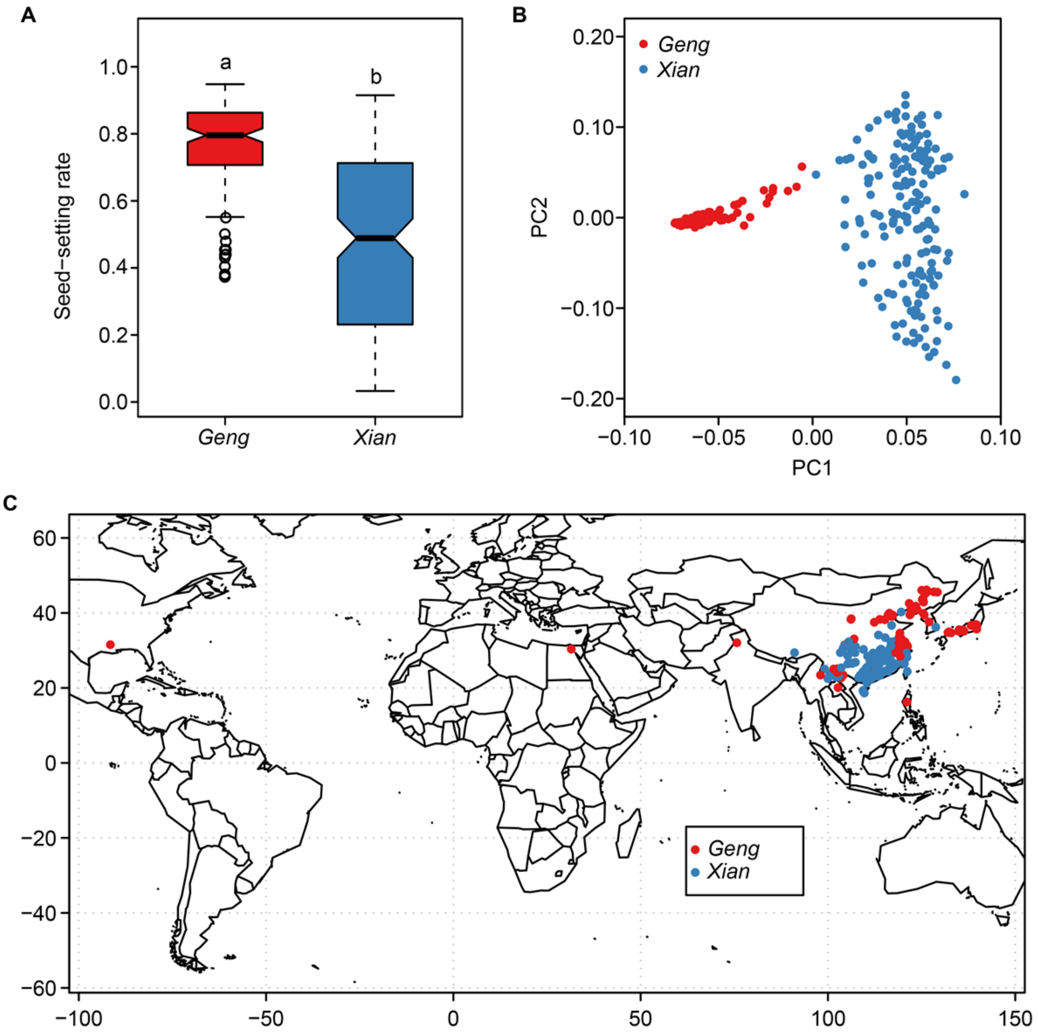

Figure S14. Characterization of 140 *Geng* and 169 *Xian* accessions from Panel 1. (A) Comparison of the seed-setting rates between subspecies. Significance of differences were determined by double-tailed Student's *t*-tests. (B) Population structure of selected *Geng* and *Xian* accessions revealed by the principal component analysis. (C) Geographic distribution of selected *Geng* and *Xian* accessions.

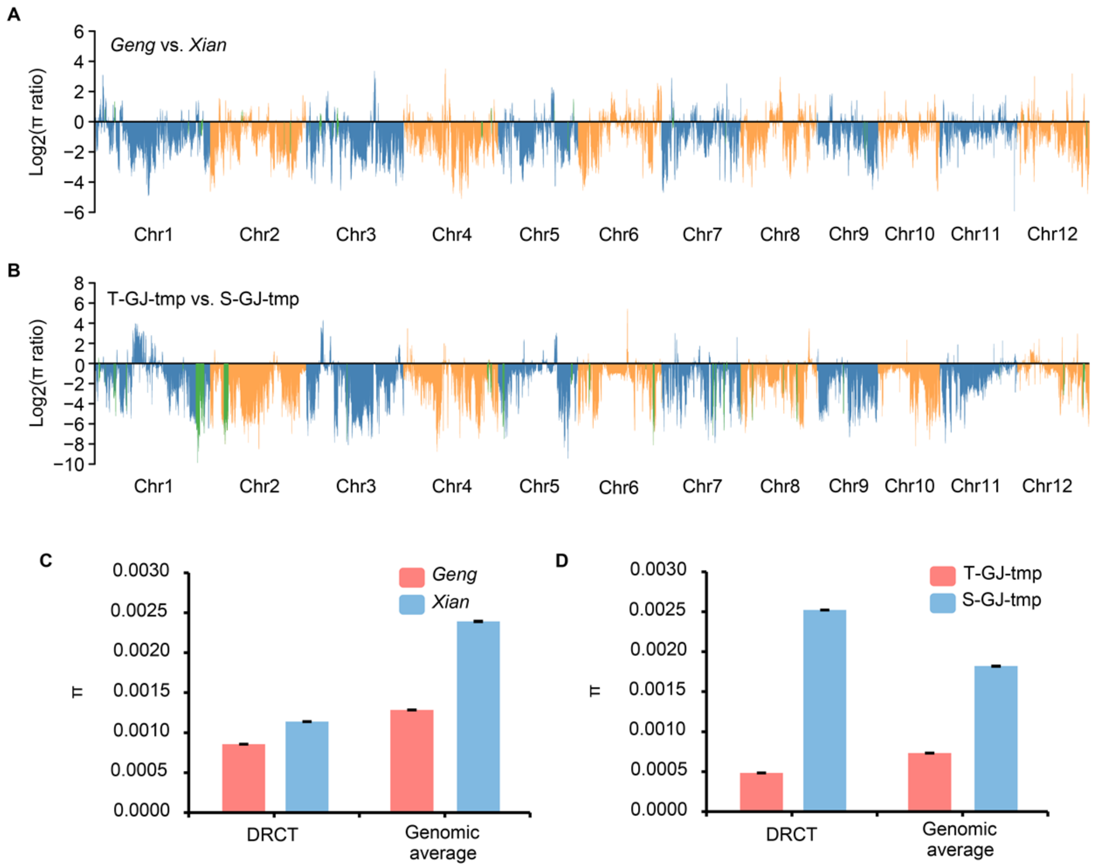

Figure S15. Nucleotide diversity of DRCT and genomic average in different populations. (A, B) Comparison of nucleotide diversity between *Geng* and *Xian* (A) and within *Geng* (B). Green columns represent the divergent regions related to cold tolerance (DRCT). (C, D) Nucleotide diversity of DRCT and genomic average in *Geng* and *Xian* (C), and in cold-tolerant temperate *Geng* (T-GJ-tmp) and cold-sensitive temperate *Geng* (S-GJ-tmp) (D).

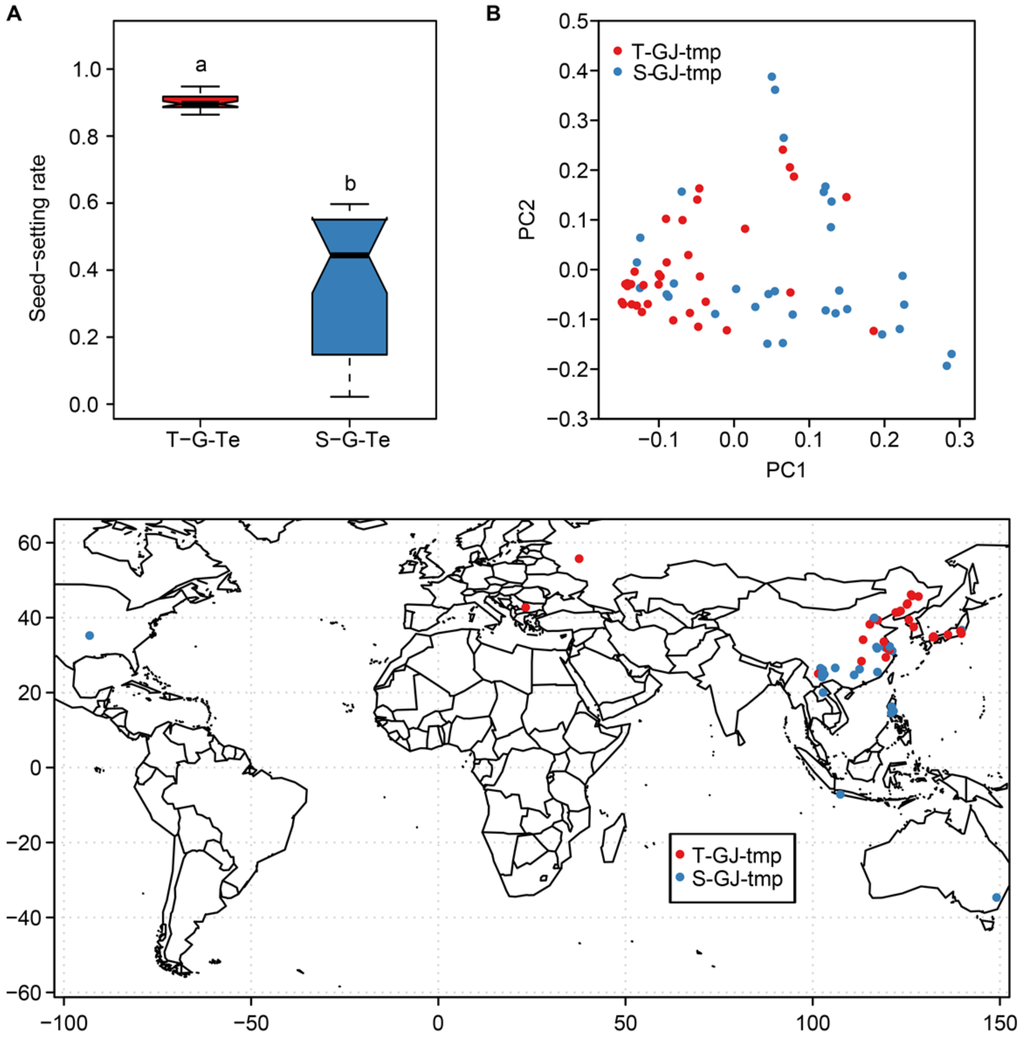

Figure S16. Characterization of 35 cold-tolerant and 32 cold-sensitive temperate *Geng* accessions from Panel 1. (A) Comparison of the seed-setting rates between selected *Geng* accessions. Significance of differences were determined by double-tailed Student's *t*-tests. (B) Population structure of selected *Geng* accessions revealed by the principal component analysis. (C) Geographic distribution of selected *Geng* accessions. T-GJ-tmp, cold-tolerant temperate *Geng*; S-GJ-tmp, cold-sensitive temperate *Geng*.

A

Genes	No. of haplotypes (Full)	P value (Full)	No. of haplotypes (*Geng*)	P value (*Geng*)	No. of haplotypes (*Xian*)	P value (*Xian*)
CTB4a	3	3.07E-03	3	1.77E-02	2	0.068
Ctb1	6	5.49E-04	3	1.74E-02	6	0.002
OsAPX1	6	2.75E-04	1	-	5	0.565
bZIP73	4	3.39E-04	1	-	3	0.553

B

Haplotype	Sub-species	promoter										No. and frequency		Seed-setting rate
		-1943	-1902	-1449	-1419	-1371	-1370	-1360	-1359	-701	-390	*Xian*	*Geng*	
Hap1	*Geng*	A	G	G	C	A	T	G	C	G	G		27(50%)	0.775
Hap3		A	T	G	T	G	T	A	T	G	A		4(7%)	0.647
Hap4		C	T	A	C	G	T	A	C	G	G		23(43%)	0.688
Hap1	*Xian*	A	G	G	C	A	T	G	C	G	G	12(15%)		0.683
Hap2		A	T	G	T	G	C	A	C	A	A	32(41%)		0.638
Hap3		A	T	G	T	G	T	A	T	G	A	19(24%)		0.559
Hap4		C	T	A	C	G	T	A	C	G	G	9(12%)		0.627
Hap5		A	T	G	T	G	T	A	C	A	A	3(4%)		0.482
Hap6		A	T	G	T	G	T	A	C	G	A	3(4%)		0.487

C

Haplotype	Sub-species	promoter						cds	No. and frequency		seed-setting rate
		-1732	-1603	-1457	-818	-712	-699	2711	*Xian*	*Geng*	
Hap1	*Geng*	C	G	T	A	A	G	C		53(98%)	0.753
Hap3		C	G	A	T	G	G	C		1(2%)	0.670
Hap1	*Xian*	C	G	T	A	A	G	C	2(3%)		0.654
Hap2		C	G	A	T	A	G	C	45(58%)		0.618
Hap3		C	G	A	T	G	G	C	10(13%)		0.524
Hap4		C	A	A	A	A	A	T	7(9%)		0.521
Hap5		C	G	A	A	A	A	C	10(13%)		0.625
Hap6		T	A	A	A	A	A	C	4(5%)		0.658

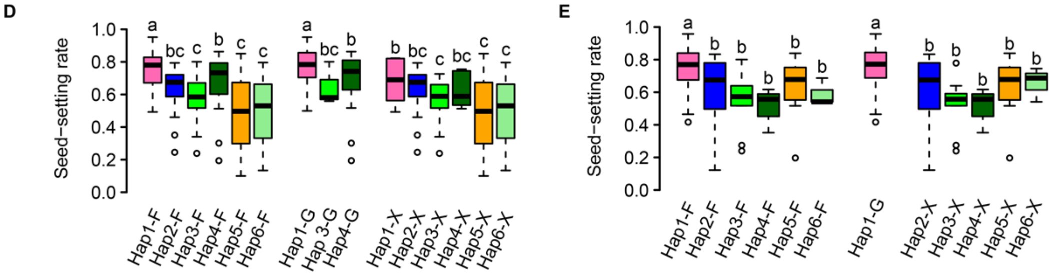

Figure S17. Association and haplotype analyses for cloned genes conferring cold tolerance at the reproductive stage in 132 accessions from Panel 2. (A) Haplotype-level association analysis for four cloned genes conferring cold tolerance at the reproductive stage. (B, C) The haplotypes of *Ctb1*(B) and *OsAPX1* (C) in 78 *Xian* and 54 *Geng* accessions. Number in parentheses represent the frequencies of different haplotypes in *Geng* and *Xian* subpopulations respectively. (D, E) Comparison of the seed-setting rates among different haplotypes of *Ctb1* (D) and *OsAPX1* (E). Haplotypes with the F, G and X suffixes indicate that the haplotypes were identified in the full population, *Geng* and *Xian* subpopulations respectively. Haplotypes filled in color are favorable haplotypes.

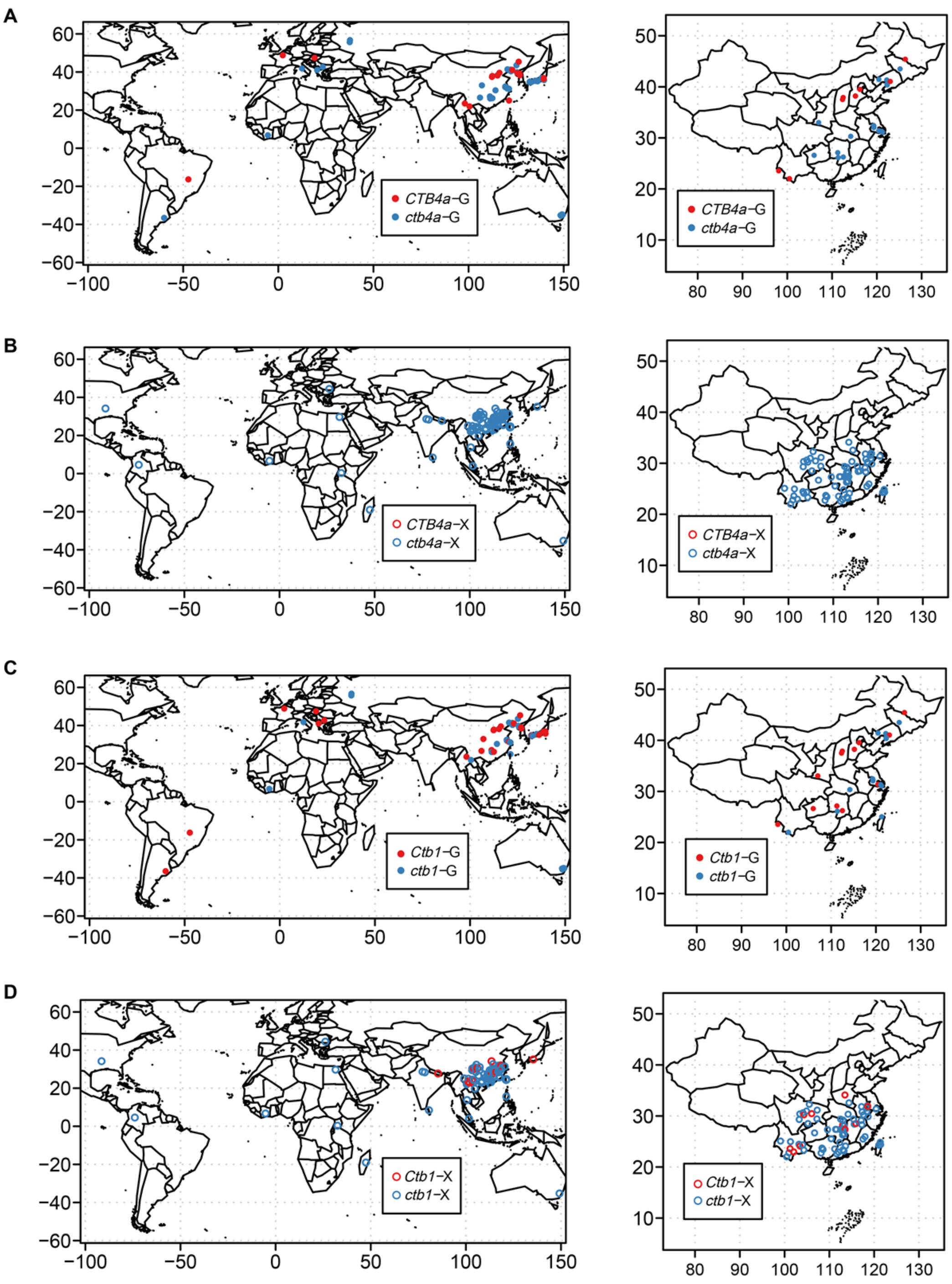

Figure S18. Allelic distributions of *CTB4a* and *Ctb1* in the world (left) and China (right). (A, B) Distribution of *CTB4a* alleles in *Geng* (A) and *Xian* (B). (C, D) Distribution of *Ctb1* alleles in *Geng* (C) and *Xian* (D). G, *Geng*; X, *Xian*.

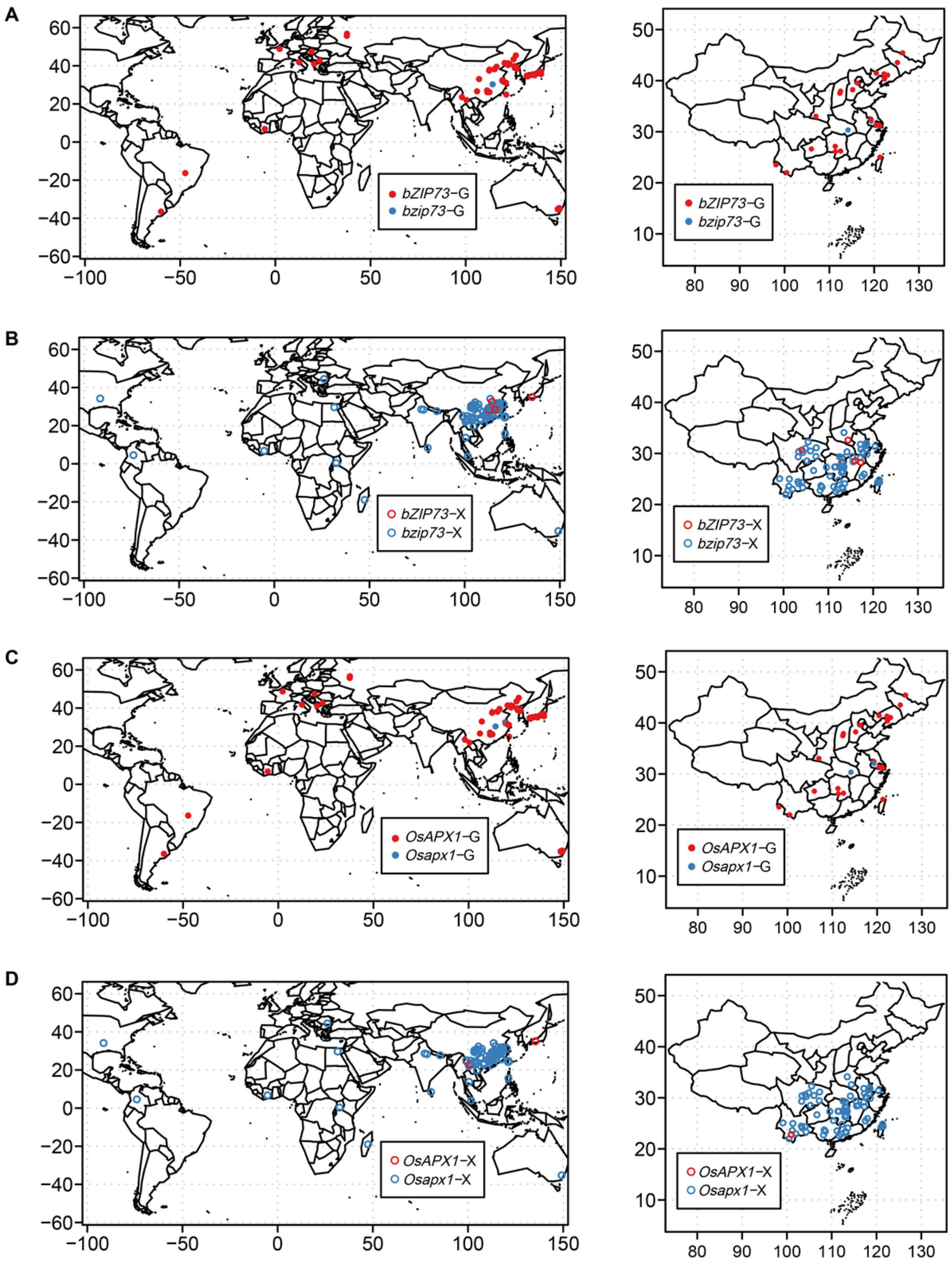

Figure S19. Allelic distributions of *bZIP73* and *OsAPX1* in the world (left) and China (right). (A, B) Distribution of *bZIP73* alleles in *Geng* (A) and *Xian* (B). (C, D) Distribution of *OsAPX1* alleles in *Geng* (C) and *Xian* (D). G, *Geng*; X, *Xian*.
